# A plasma protein signature associated with cognitive function in men without severe cognitive impairment

**DOI:** 10.1186/s13195-023-01294-7

**Published:** 2023-09-01

**Authors:** Kanika Mehta, Mohammadreza Mohebbi, Julie A. Pasco, Lana J. Williams, Sophia X. Sui, Ken Walder, Boon Lung Ng, Veer Bala Gupta

**Affiliations:** 1 Deakin University, IMPACT – The Institute for Mental and Physical Health and Clinical Translation, School of Medicine, Geelong, VIC 3216 Australia; 2 Baker Heart and Diabetes Institute, Melbourne, VIC Australia; 3 Biostatistics Unit, Faculty of Health, Deakin University, Burwood, VIC Australia; 4 Department of Medicine-Western Health, The University of Melbourne, St Albans, VIC Australia; 5 Department of Epidemiology and Preventive Medicine, Monash University, Prahran, VIC Australia; 6 Barwon Health, Geelong, VIC Australia; 7 Department of Geriatric Medicine, Barwon Health, Geelong, VIC Australia

**Keywords:** Cognitive function, Alzheimer’s disease, Proteomic analysis, Genotyping, Risk factors

## Abstract

**Background:**

A minimally invasive blood-based assessment of cognitive function could be a promising screening strategy to identify high-risk groups for the incidence of Alzheimer’s disease.

**Methods:**

The study included 448 cognitively unimpaired men (mean age 64.1 years) drawn from the Geelong Osteoporosis Study. A targeted mass spectrometry-based proteomic assay was performed to measure the abundance levels of 269 plasma proteins followed by linear regression analyses adjusted for age and *APOE* ε4 carrier status to identify the biomarkers related to overall cognitive function. Furthermore, two-way interactions were conducted to see whether Alzheimer’s disease-linked genetic variants or health conditions modify the association between biomarkers and cognitive function.

**Results:**

Ten plasma proteins showed an association with overall cognitive function. This association was modified by allelic variants in genes *ABCA7*, *CLU*, *BDNF* and *MS4A6A* that have been previously linked to Alzheimer’s disease. Modifiable health conditions such as mood disorders and poor bone health, which are postulated to be risk factors for Alzheimer’s disease, also impacted the relationship observed between protein marker levels and cognition. In addition to the univariate analyses, an 11-feature multianalyte model was created using the least absolute shrinkage and selection operator regression that identified 10 protein features and age associated with cognitive function.

**Conclusions:**

Overall, the present study revealed plasma protein candidates that may contribute to the development of a blood-based screening test for identifying early cognitive changes. This study also highlights the importance of considering other risk factors in elucidating the relationship between biomarkers and cognition, an area that remains largely unexplored.

**Supplementary Information:**

The online version contains supplementary material available at 10.1186/s13195-023-01294-7.

## Background

There is a substantial rise in the number of people living with Alzheimer’s disease (AD), which is one of the leading causes of mortality worldwide. High prevalence and associated disability have necessitated a focus on investigating its preclinical phase, where biochemical changes are thought to begin 15–20 years prior to the presentation of clinical symptoms [1]. This is known as the asymptomatic stage where a screening test might be useful in identifying individuals at risk of developing AD. Currently, the most promising sets of biomarkers for identifying preclinical AD constitute amyloid β (Aβ) or tau positron emission tomography (PET) imaging and their measurement in the cerebro spinal fluid (CSF) [1–3]. However, these approaches are limited in their use as population screening tools and unlikely to attract high participation rates as PET scans are expensive and only available in specialised centres, while the collection of CSF involves an invasive lumbar puncture procedure that carries the risk of adverse effects. The use of blood biomarkers instead circumvents the above issues and is potentially a more useful screening tool for the general population. Despite this, there is a paucity of studies investigating blood biomarkers associated with cognitive function among non-demented individuals as the majority focus on the identification of dementia biomarkers. There is also a risk of misclassification with the existing plasma biomarkers for amyloid pathology, for example, the Aβ42/Aβ40 ratio, precluding their implementation in routine clinical settings [4]. Another challenge with the use of blood biomarkers in clinical settings is the lack of reproducibility, which possibly could be due to the high complexity and wide dynamic range of protein abundances in the blood as well as limited sensitivity offered by immunoassays. However, advancements in the area of targeted mass spectrometry have resulted in highly sensitive and specific multiplexed methods that can accurately quantify low abundant protein markers [5].

The lack of reproducible results can also be due to the multifactorial nature of AD pathology [6]. AD is a complex condition that involves an interplay between several genetic and environmental factors, which are often not considered in biomarker studies [7]. For instance, genetic predisposition has a substantial role in the development of AD, and individuals who are carriers of allelic variants linked to AD risk may have an altered biomarker profile as compared to those who do not harbour the risk alleles. We hypothesised that genetic variants previously linked to AD risk may modify the relationship observed between protein markers and cognitive function. We also expected some of the modifiable physical and mental health conditions associated with AD to have a similar impact on the relationship between protein markers and cognition. Several studies including systematic reviews and meta-analyses have postulated depression and poor bone health to be risk factors for AD or general cognitive decline [8–12]. In light of AD’s complex aetiology, it is important to account for genetic variants and health conditions related to AD to obtain reliable estimates of biomarker levels. Therefore, the primary aim of our study was to identify blood protein markers associated with cognitive function among a population cohort of 448 healthy male participants using a high-throughput mass spectrometric platform. Furthermore, we investigated two-way interactions to see whether AD-linked genetic variants or health conditions modify the association between biomarkers and cognitive function.

## Methods

### Study cohort, assessment procedures and sample collection

The present study analysed data and blood samples collected from men participating in the Geelong Osteoporosis Study (GOS), an ongoing, prospective population-based study. In brief, age-stratified samples of men and women were selected at random from electoral rolls for the Barwon Statistical Division in south-eastern Australia [13]. The inclusion criteria were a listing on the electoral rolls for the Barwon Statistical Division and residence in the area for a minimum of six months. A total of 1540 men were recruited from 2001 to 2006 (67% participation) and returned for follow-up 5 and 15 years post-recruitment. This study includes a cross-sectional analysis of data and blood samples collected from 448 men during the 15-year follow-up phase (2016–2020). Further information on participant selection is provided in Additional file 1: Fig. S1. As cognitive testing using the CogState Brief Battery (CBB) is currently underway for GOS women, their data could not be included in the present study. Participants were mostly Caucasian (~ 98%). They provided information on their lifestyle and demographic characteristics in addition to undergoing mental and physical health assessments.

Cognitive function was evaluated using a computer-based neuropsychology battery, the CBB, which has been described previously [14–16]. The CBB requires participants to respond to stimuli cards as a part of detection (DET), identification (IDN), one-card learning (OCL) and one-back (OBK) tasks that assess cognitive performance across four domains, namely psychomotor function, visual identification, recognition memory and working memory, respectively. Both a practice trial and a real test were included for each task. The tasks were completed by participants in a quiet room accompanied by a researcher. For the tasks DET, IDN and OBK, scores were calculated by measuring the time (milliseconds) taken to answer correctly, which was then normalised using a log_10_ transformation. For the OCL task, scores were calculated based on the accuracy of participant response and normalised using an arcsine square-root transformation. Furthermore, scores for the overall cognitive function (OCF, unitless) were determined by combining the primary measures in the four domains. The present analysis only includes scores from the overall cognitive function, for which higher scores indicate better performance. In addition, participants attended the Mini-Mental State Examination (MMSE) that assessed their overall cognitive function [17]. However, due to inherent limitations associated with MMSE, only OCF scores from the CBB were used in the analysis [18].

**Fig. 1:**
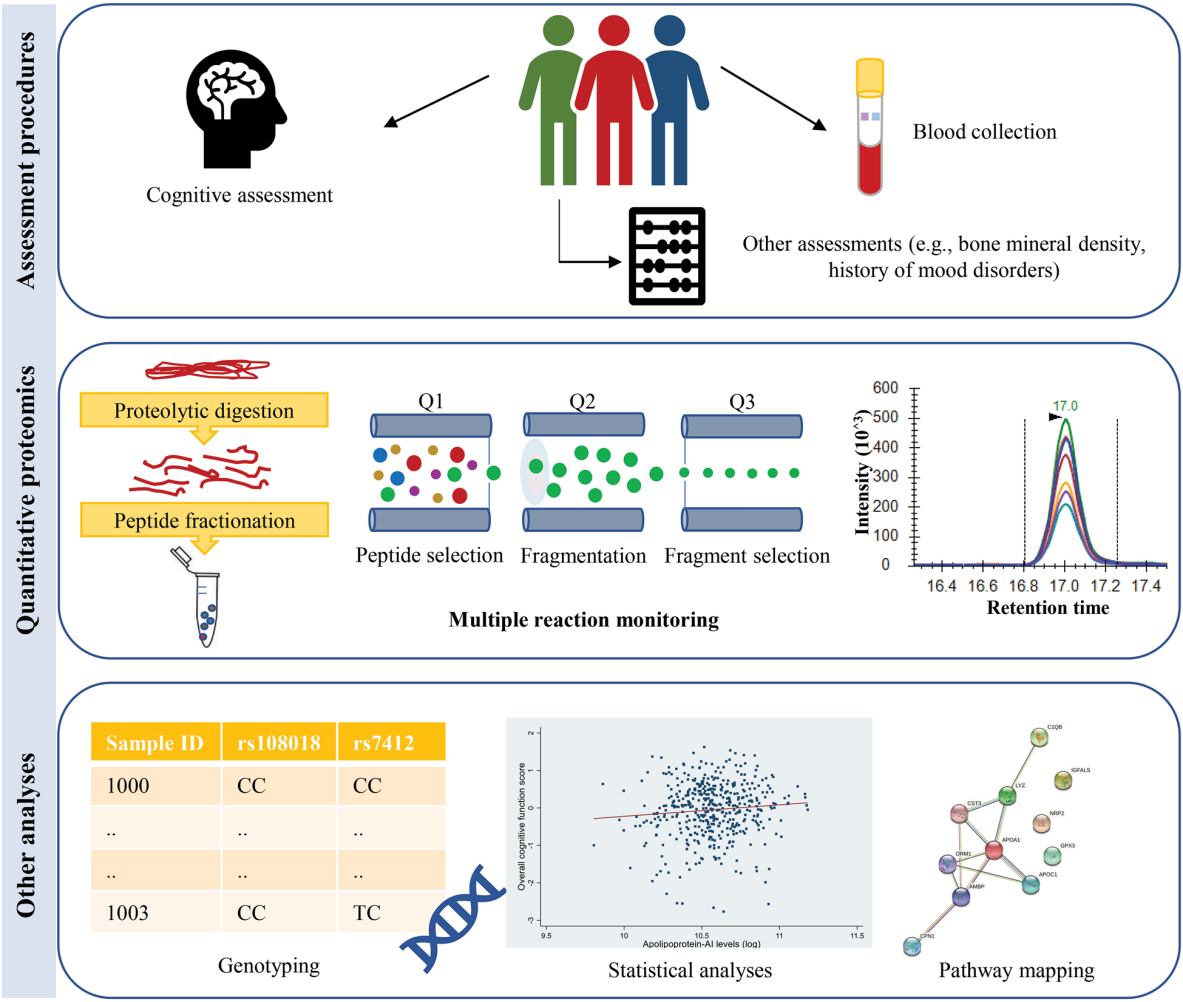
Schematic representation of the workflow

Details on sociodemographic variables such as education, smoking and domestic partnership status were acquired from self-reports. Education was defined as a nominal factor based on secondary education completion. Similarly, domestic partnership status was defined as living with a partner (coded “1”) or not (coded “0”). Participants who reported smoking at least one cigarette per day were defined as current smokers. The Structured Clinical Interview for *Diagnostic and Statistical Manual of Mental Disorders, Fourth Edition*, Non-Patient Edition (SCID-I/NP) was used to determine the current mood disorders, as described previously [19]. Bone mineral density (BMD; g/cm^2^) was measured at the total hip, femoral neck and lumbar spine (posterior-anterior projection, L2–L4) using dual-energy X-ray absorptiometry (GE Lunar, Prodigy Pro, Madison, WI, USA) [20, 21]. Furthermore, participants reported whether they suffered a major osteoporotic fracture in the last 20 years that included hip, vertebral, wrist or humerus fractures and confirmed using radiology reports as previously described [22]. Fractures that occurred in a motor vehicle accident were not considered. Blood plasma samples were collected after overnight fasting in EDTA tubes at the Australian Clinical Labs and stored at − 80 °C until use. Cognitive assessments and other health measurements were conducted by trained technicians who were not involved in blood biomarker measurements or data analysis. A schematic representation of the workflow is shown in Fig. 1.

### Liquid chromatography-mass spectrometry (LC-MS)-based proteomic analysis

The plasma samples were shipped on dry ice to the University of Victoria – Genome British Columbia Proteomics Centre (BC, Canada) where they were analysed for a panel of 269 proteins. These are common plasma proteins that belong to physiological processes such as inflammation, lipid transport, signalling, oxidative stress and immune response. A complete list of plasma protein markers included in the assay is provided in Additional file 1: Table S1. A peptide-based targeted quantitation was conducted using multiple reaction monitoring assays following the Clinical Proteomic Tumor Analysis Consortium guidelines for assay development (https://assays.cancer.gov/). Tryptic peptides were selected to serve as molecular surrogates for the 269 target proteins according to a series of peptide selection rules (e.g. unique sequence, devoid of oxidisable residues and previous detectability in plasma samples). To help compensate for a matrix-induced suppression or variability in LC-MS performance, ^13^C/^15^N-labelled peptide analogues were used as internal standards. All peptides were synthesised via Fmoc chemistry, purified through reversed-phase high-performance liquid chromatography and characterised via amino acid analysis and capillary zone electrophoresis. A detailed protocol has been described previously [23]. The plasma proteolytic digests were analysed using a triple quadrupole mass spectrometer (Agilent 6495) in the positive ion mode. The data were visualised and examined using the Skyline Quantitative Analysis software (version 21.0.9.139, University of Washington). This involved peak inspection to ensure accurate selection, integration and uniformity in terms of peak shape and retention time. After defining a small number of criteria (i.e. 1/*x* regression weighting, < 20% deviation in the QC’s level’s accuracy), a standard curve was used to calculate the peptide concentration in fmol/μL of plasma.

### DNA extraction and genotyping

Total genomic DNA was isolated from buffy coats using the QIAamp® DNA Mini Kit (Qiagen, Hilden, Germany) as per the manufacturer’s instructions. The DNA samples were genotyped for the following 11 single nucleotide polymorphisms (SNPs): rs429358 (apolipoprotein E; *APOE* ε4), rs7412 (apolipoprotein E; *APOE* ε2), rs744373 (bridging integrator 1; *BIN1*), rs11136000 (clusterin; *CLU*), rs3764650 (ATP-binding cassette subfamily A member 7; *ABCA7*), rs3818361 (complement receptor 1; *CR1*), rs3851179 (phosphatidylinositol-binding clathrin assembly protein; *PICALM*), rs3865444 (cluster of differentiation 33; *CD33*), rs610932 (membrane spanning 4-domains A6A; *MS4A6A*), rs6265 (brain-derived neurotrophic factor; *BDNF*) and rs9349407 (CD2-associated protein; *CD2AP*) at the Australian Genome Research Facility, Brisbane, using the Agena Bioscience MassARRAY® platform. These SNPs are associated with late-onset AD and were selected based on a thorough literature review including the meta-analysis results from the AlzGene database [24]. The carrier status was defined by the presence of at least one copy of the risk allele. No departure from the Hardy-Weinberg equilibrium was detected. The genetic data analysed in this study are provided in Additional file 1: Table S2.

### Statistical analyses

Data cleaning steps were applied to the proteomic data prior to the analyses. For each protein, concentration values outside the limit of quantitation were treated as missing, following which protein analytes with greater than 10% missing values were dropped [3, 25]. For the remaining 125 proteins with more than 90% of data available, missing values were imputed by assigning the lower limit of quantitation/2 value [3]. Furthermore, the protein concentrations were transformed to a natural logarithm to achieve normal distribution. Individual linear regression analyses were conducted to investigate the association between overall cognitive function and protein concentrations. Age and *APOE* ε4 carrier status were included as confounders in the regression model. As this was an exploratory analysis, correction for multiple testing was not applied.

Next, two-way interactions between proteins that showed a significant association with cognitive function and nine AD-linked genetic polymorphisms [rs744373 (*BIN1*), rs11136000 (*CLU*), rs3764650 (*ABCA7*), rs3818361 (*CR1*), rs3851179 (*PICALM*), rs3865444 (*CD33*), rs610932 (*MS4A6A*), rs6265 (*BDNF*) and rs9349407 (*CD2AP*)] were explored (in separate regression analyses) to see whether the latter affects the relationship between cognition and protein markers. The interaction analyses were adjusted for age and *APOE* ε4 status. Similar interaction analyses were performed to investigate whether comorbidities such as mood disorders and bone loss affect the relationship observed between cognitive function and protein markers. All statistical analyses were performed using Stata/SE 17.0.

### Multianalyte model building and functional mapping of proteins

While the univariate analysis described above revealed associations between a single protein and cognitive function, classifiers and machine learning methods enable the identification of a panel of features that relate to the condition only when considered in combination with one another. Thus, a linear multianalyte model was built using the least absolute shrinkage and selection operator (LASSO) method. A total of 127 features were selected that included 125 protein markers, age and *APOE* ε4 carrier status. A 10-fold cross-validation method was used to develop the model. The optimal regularisation parameter (lambda) was estimated through the 10-fold cross-validation process to maximise the out-of-sample *R*^2^ and minimise the mean prediction error. The analysis was performed using Stata/SE 17.0.

Furthermore, complex interactions among proteins identified through LASSO were investigated using bioinformatic tools such as NetworkAnalyst 3.0 (http://www.networkanalyst.ca/) [26] and STRING version 11.5 (https://string-db.org/) [27]. The list of UniProt protein accession numbers was uploaded to generate the protein-protein interaction networks. In order to identify the perturbed pathways, pathway mapping was performed using Reactome.org (www.reactome.org) [28].

## Results

Participant characteristics are presented in Table 1. The study participants had a mean age of 64.1 years (SD 13.3), and more than three-quarters had completed secondary education (75.5%) and were living with a partner (81.5%). The average MMSE score of the participants was 28.6 (SD 1.7), and only nine had an MMSE score less than 24, which suggests cognitive impairment. Among them, one participant scored 19, another scored 21, two scored 22 and the remaining five scored 23 on MMSE. Overall, 25.9% of all participants carried the *APOE* ε4 risk allele.

**Table 1:** Demographic characteristics of the study participants (*n* = 448). Data are presented as mean (SD) or *n* (%)

**Variable**		**Mean (SD)/*n* (%)**
Age [years], mean (SD)		64.1 (13.3)
Education^a^, *n* (%)	Secondary education completed	338 (75.5)
	Secondary education not completed	109 (24.3)
Domestic partnership status, *n* (%)	Living with a partner	365 (81.5)
	Not living with a partner	83 (18.5)
Current smoker, *n* (%)	Yes	30 (6.7)
	No	418 (93.3)
*APOE* ε4 carriage^a^, *n* (%)	Yes	116 (25.9)
	No	323 (72.1)
Current mood disorder^a^, *n* (%)	Yes	22 (4.9)
	No	37 (8.3)
Femoral neck BMD [g/cm^2^], mean (SD)		1.0 (0.1)
Total hip BMD [g/cm^2^], mean (SD)		1.1 (0.1)
Spine BMD [g/cm^2^], mean (SD)		1.3 (0.2)
Occurrence of a major osteoporotic fracture in the last 20 years, *n* (%)	Yes	33 (7.4)
	No	415 (92.6)
MMSE, mean (SD)		28.6 (1.7)
CBB-IDN, mean (SD)		2.7 (0.1)
CBB-DET, mean (SD)		2.5 (0.1)
CBB-OBK, mean (SD)		2.9 (0.1)
CBB-OCL, mean (SD)		1.0 (0.1)
CBB-OCF, mean (SD)		− 0.01 (0.7)

﻿^a^Excludes missing values

### Association between plasma protein levels and overall cognitive function

Preliminary data cleaning steps resulted in 125 proteins that were analysed. Of these, 10 proteins, namely apolipoprotein A-I, apolipoprotein C-I, apolipoprotein M, carboxypeptidase N catalytic chain, complement C1q subcomponent subunit B, complement C1q subcomponent subunit C, glutathione peroxidase 3, lysozyme C, neuropilin-2/cystatin-C and alpha-1-microglobulin were found to be significantly associated with cognitive function (Table 2). Among these, the biggest effect size was observed for the protein apolipoprotein A-I; with every 1% rise in plasma apolipoprotein A-I levels, the average cognitive function score increased by 0.004 units.

**Table 2:** Results from the linear regression analysis investigating the association between plasma protein levels and overall cognitive function

**Protein**	***B***_**coeff**_	**95% CI**	***t*-value**	***p*-value**	^a^**Partial eta-squared**
Apolipoprotein A-I	0.38	0.11, 0.65	2.80	0.005	0.02
Apolipoprotein C-I	0.18	0.04, 0.32	2.46	0.014	0.01
Apolipoprotein M	0.23	< 0.01, 0.47	1.97	0.050	0.01
Carboxypeptidase N catalytic chain	0.22	< 0.01, 0.44	2.01	0.046	0.01
Complement C1q subcomponent subunit B	− 0.24	− 0.47, − 0.01	− 2.05	0.040	0.01
Complement C1q subcomponent subunit C	− 0.22	− 0.44, − 0.01	− 2.01	0.045	0.01
Glutathione peroxidase 3	0.28	0.04, 0.51	2.29	0.022	0.01
Lysozyme C	− 0.18	− 0.36, − 0.01	− 2.06	0.040	0.01
Neuropilin-2/cystatin-C	− 0.21	− 0.41, − 0.01	− 2.02	0.044	0.01
Alpha-1-microglobulin/bikunin precursor	− 0.19	− 0.373, − 0.004	− 2.01	0.045	0.01

The protein concentrations were transformed into a natural log, and the analyses were adjusted for age and *APOE* ε4 carrier status

^a^An eta-squared value of < 0.02 is considered a small effect size

### Interaction between protein markers and genetic variants (linked to AD) for predicting cognitive function

**Fig. 2:**
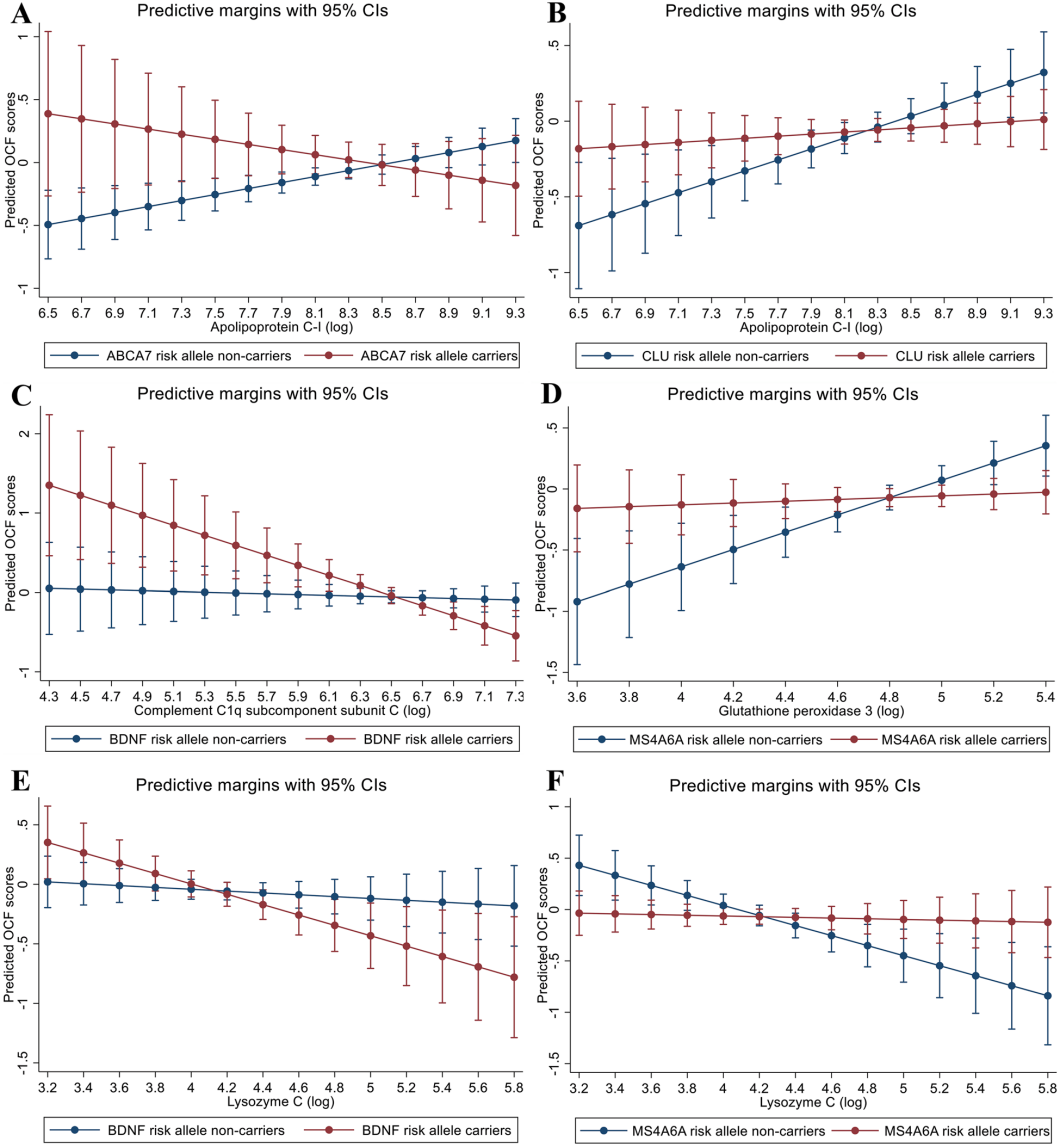
Predictive margins plots displaying interactions of protein markers with AD-related genetic variants for predicting overall cognitive function. **A** Interaction of apolipoprotein C-I with *ABCA7* risk allele. **B** Interaction of apolipoprotein C-I with *CLU* risk allele. **C** Interaction of complement C1q subcomponent subunit C with *BDNF* risk allele. **D** Interaction of glutathione peroxidase 3 with *MS4A6A* risk allele. **E** Interaction of lysozyme C with *BDNF* risk allele. **F** Interaction of lysozyme C with *MS4A6A* risk allele

Four genetic variants: rs3764650 (*ABCA7*), rs11136000 (*CLU*), rs6265 (*BDNF*) and rs610932 (*MS4A6A*) that have been previously associated with AD risk, modified the relationship between protein markers and cognitive function (Table 3 and Fig. 2). For instance, individuals with increasing plasma apolipoprotein C-I levels showed a higher score for cognitive function; however, among those carrying the *ABCA7* risk allele, increasing apolipoprotein C-I levels were associated with poorer cognitive function (Fig. 2A). Among the risk allele carriers, with every 1% rise in plasma apolipoprotein C-I levels, the average cognitive function score decreased by 0.004 units. A similar pattern was observed for apolipoprotein C-I’s interaction with the risk allele belonging to the *CLU* gene (Fig. 2B). Increasing plasma apolipoprotein C-I levels were associated with a steep rise in cognitive function among individuals who did not carry the risk allele; however, this was greatly diminished among carriers, for whom a minimal change in cognitive function scores was observed with increasing apolipoprotein C-I levels.

**Table 3:** Results from the interaction analyses between plasma protein levels and genetic variants for predicting overall cognitive function

**Exposure**	***B***_**coeff**_	**95% CI**	***t*-value**	***p*-value**	^a^**Partial eta-squared**
Interaction between apolipoprotein C-I and rs3764650 (*ABCA7*)					
Apolipoprotein C-I	0.17	0.03, 0.32	2.42	0.016	0.01
*ABCA7*_carrier	0.10	− 0.06, 0.26	1.27	0.204	< 0.01
Apolipoprotein C-I#*ABCA7*_carrier	− 0.44	− 0.83, − 0.05	− 2.24	0.026	0.01
Interaction between apolipoprotein C-I and rs11136000 (*CLU*)					
Apolipoprotein C-I	0.17	0.03, 0.31	2.37	0.018	0.01
*CLU*_carrier	− 0.001	− 0.12, 0.12	− 0.02	0.986	< 0.01
Apolipoprotein C-I#*CLU*_carrier	− 0.29	− 0.58, − 0.01	− 2.00	0.046	0.01
Interaction between complement C1q subcomponent subunit C and rs6265 (*BDNF*)					
Complement C1q subcomponent subunit C	− 0.22	− 0.438, − 0.005	− 2.01	0.045	0.01
*BDNF*_carrier	− 0.01	− 0.13, 0.12	− 0.10	0.919	< 0.01
Complement C1q subcomponent subunit C#*BDNF*_carrier	− 0.58	− 1.06, − 0.11	− 2.43	0.015	0.01
Interaction between glutathione peroxidase 3 and rs610932 (*MS4A6A*)					
Glutathione peroxidase 3	0.28	0.04, 0.51	2.29	0.023	0.01
*MS4A6A*_carrier	− 0.02	− 0.15, 0.10	− 0.38	0.707	< 0.01
Glutathione peroxidase 3#*MS4A6A*_carrier	− 0.64	− 1.12, − 0.15	− 2.56	0.011	0.01
Interaction between lysozyme C and rs6265 (*BDNF*)					
Lysozyme C	− 0.19	− 0.36, − 0.01	− 2.06	0.040	0.01
*BDNF*_carrier	− 0.01	− 0.14, 0.11	− 0.23	0.815	< 0.01
Lysozyme C#*BDNF*_carrier	− 0.36	− 0.71, − 0.01	− 2.00	0.047	0.01
Interaction between lysozyme C and rs610932 (*MS4A6A*)					
Lysozyme C	− 0.18	− 0.36, − 0.01	− 2.05	0.041	0.01
*MS4A6A*_carrier	− 0.02	− 0.15, 0.10	− 0.39	0.696	< 0.01
Lysozyme C#*MS4A6A*_carrier	0.45	0.12, 0.79	2.65	0.008	0.02

The protein concentrations were transformed into a natural log, and the analyses were adjusted for age and *APOE* ε4 carrier status. The individual main effects are from a model without the interaction term

^a^An eta-squared value of < 0.02 is considered a small effect size

For the interaction between protein complement C1q subcomponent subunit C and *BDNF* risk allele, a weak negative association was observed between the protein level and cognitive function among non-carriers (Fig. 2C). Although the directionality of association remained the same among risk allele carriers, the decline in cognitive function was greater.

### Interaction between protein markers and health conditions such as mood disorders and bone health for predicting cognitive function

**Fig. 3:**
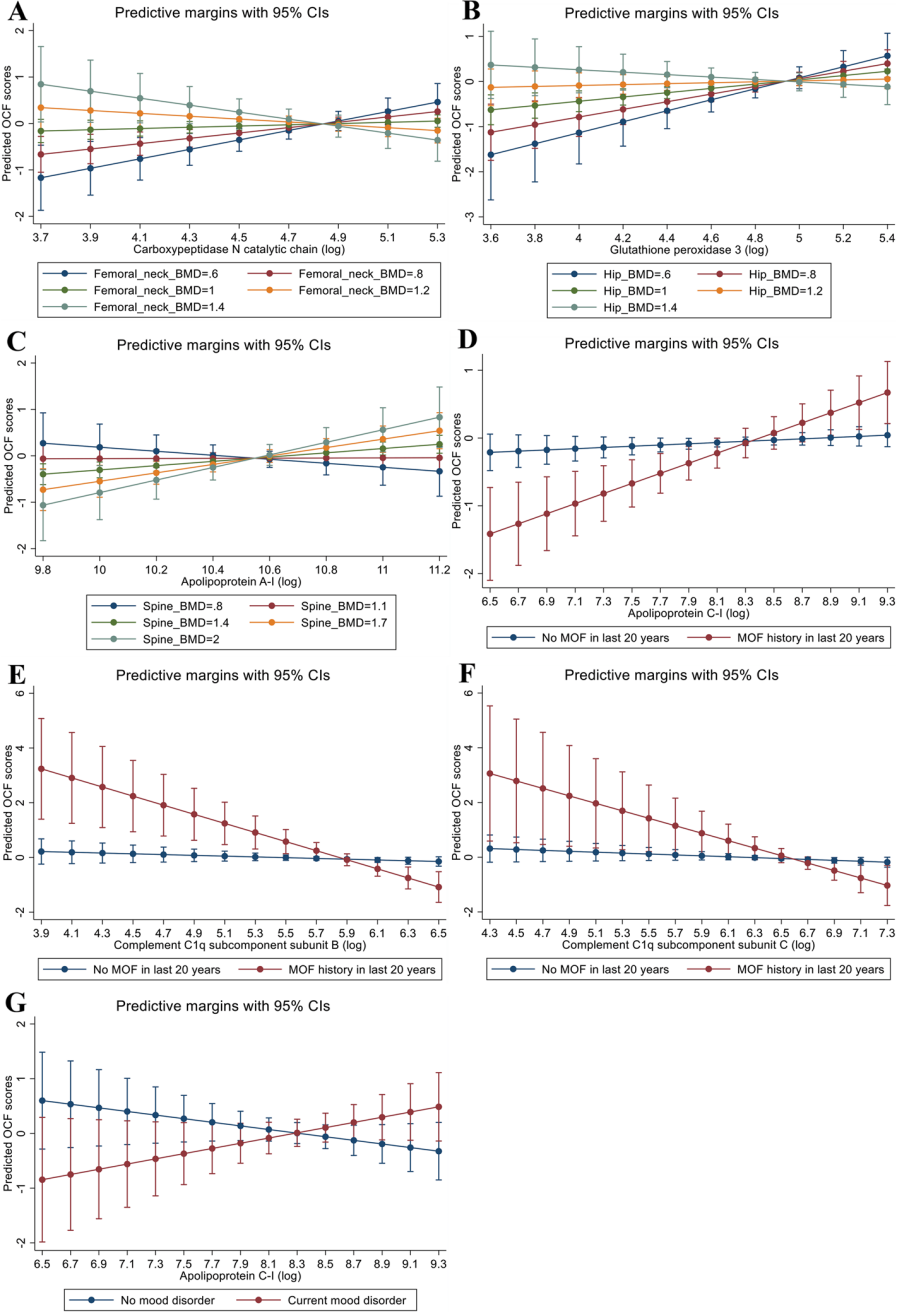
Predictive margin plots displaying the interactions of protein markers with AD-related health conditions for predicting overall cognitive function. **A** Interaction of carboxypeptidase N catalytic chain with femoral neck BMD. **B** Interaction of glutathione peroxidase 3 with total hip BMD. **C** Interaction of apolipoprotein A-I with spine BMD. **D** Interaction of apolipoprotein C-I with major osteoporotic fractures in the last 20 years. **E** Interaction of complement C1q subcomponent subunit B with major osteoporotic fractures in the last 20 years. **F** Interaction of complement C1q subcomponent subunit C with major osteoporotic fractures in the last 20 years. **G** Interaction of apolipoprotein C-I with a current mood disorder

Six proteins: carboxypeptidase N catalytic chain, glutathione peroxidase 3, apolipoprotein A-I, apolipoprotein C-I, complement C1q subcomponent subunit B and complement C1q subcomponent subunit C, showed a significant interaction with mood disorders or bone health-related variables for predicting overall cognitive function (Table 4 and Fig. 3). An overall negative interaction was observed between carboxypeptidase N catalytic chain and bone mineral density at the femoral neck as shown in Table 4. A closer inspection of the predictive margin plots revealed that for femoral neck BMD values up to 1.0, a positive association between carboxypeptidase N catalytic chain and cognitive function existed (Fig. 3A). However, for BMD values beyond 1.0, increasing carboxypeptidase N catalytic chain was associated with a decline in cognitive function. An opposite trend was seen for the relationship between apolipoprotein A-I levels and cognitive function as modified by spine BMD (Fig. 3C). For spine BMD values up to 1.1, there was a negative association between apolipoprotein A-I levels and cognitive function that changed to a positive association for higher BMD values. Other bone health-related variables included total hip BMD and major osteoporotic fractures in the last 20 years, which also modified the association between some of the protein markers and cognitive function. In addition to variables relating to bone health, the presence of a mood disorder impacted the biomarker findings. The interaction between protein markers and mood disorder was examined for only 58 participants. Among individuals with a current diagnosis of a mood disorder, increasing apolipoprotein C-I levels correlated with higher cognitive function, while an inverse pattern was detected for people without a mood disorder (Fig. 3G).

**Table 4:** Results from the interaction analyses between plasma protein levels and variables related to bone health and mood disorder for predicting overall cognitive function

**Exposure**	***B***_**coeff**_	**95% CI**	***t*-value**	***p*-value**	^a^**Partial eta-squared**
Interaction between carboxypeptidase N catalytic chain and femoral neck BMD					
Carboxypeptidase N catalytic chain	0.20	− 0.02, 0.42	1.81	0.072	0.01
Femoral neck BMD	0.19	− 0.29, 0.66	0.78	0.434	< 0.01
Carboxypeptidase N catalytic chain#Femoral neck BMD	− 2.21	− 3.86, − 0.57	− 2.65	0.008	0.02
Interaction between glutathione peroxidase 3 and total hip BMD					
Glutathione peroxidase 3	0.36	0.12, 0.60	2.93	0.004	0.02
Total hip BMD	0.23	− 0.19, 0.66	1.08	0.282	< 0.01
Glutathione peroxidase 3#Total hip BMD	− 1.86	− 3.51, − 0.21	− 2.21	0.027	0.01
Interaction between apolipoprotein A-I and spine BMD					
Apolipoprotein A-I	0.38	0.11, 0.65	2.73	0.007	0.02
Spine BMD	0.03	− 0.28, 0.35	0.20	0.840	< 0.01
Apolipoprotein A-I#Spine BMD	1.49	0.08, 2.89	2.08	0.038	0.01
Interaction between apolipoprotein C-I and major osteoporotic fractures in the last 20 years					
Apolipoprotein C-I	0.18	0.04, 0.32	2.47	0.014	0.01
MOF_20yrs	− 0.08	− 0.30, 0.15	− 0.66	0.510	< 0.01
Apolipoprotein C-I#MOF_20yrs	0.65	0.25, 1.06	3.19	0.002	0.02
Interaction between complement C1q subcomponent subunit B and major osteoporotic fractures in the last 20 years					
Complement C1q subcomponent subunit B	− 0.24	− 0.470, − 0.005	− 2.00	0.046	0.01
MOF_20yrs	− 0.05	− 0.28, 0.18	− 0.45	0.654	< 0.01
Complement C1q subcomponent subunit B#MOF_20yrs	− 1.52	− 2.45, − 0.59	− 3.21	0.001	0.02
Interaction between complement C1q subcomponent subunit C and major osteoporotic fractures in the last 20 years					
Complement C1q subcomponent subunit C	− 0.22	− 0.434, 0.001	− 1.95	0.051	0.01
MOF_20yrs	− 0.05	− 0.28, 0.18	− 0.42	0.674	< 0.01
Complement C1q subcomponent subunit C#MOF_20yrs	− 1.20	− 2.28, − 0.12	− 2.19	0.029	0.01
Interaction between apolipoprotein C-I and a current mood disorder					
Apolipoprotein C-I	− 0.02	− 0.42, 0.38	− 0.09	0.925	< 0.01
Current mood disorder	0.03	− 0.30, 0.36	0.19	0.852	< 0.01
Apolipoprotein C-I#Current mood disorder	0.81	0.05, 1.56	2.15	0.036	0.08

The protein concentrations were transformed to a natural log, and the analyses were adjusted for age and *APOE* ε4 carrier status. The individual main effects are from a model without the interaction term. MOF_20yr refers to the occurrence of a major osteoporotic fracture in the last 20 years

^a^An eta-squared value of < 0.02 is considered a small effect size

### Variable selection and multimarker model building using LASSO

An 11-feature multimarker panel was identified through LASSO in the following order of importance: apolipoprotein A-I, neuropilin-2/cystatin-C, apolipoprotein C-I, alpha-1-microglobulin/bikunin precursor, complement C1q subcomponent subunit B, insulin-like growth factor-binding protein complex acid labile subunit, glutathione peroxidase 3, age, alpha-1-acid glycoprotein 1, lysozyme C and carboxypeptidase N catalytic chain (Additional file 1: Fig. S2). The cross-validated LASSO model had a mean squared error of 0.38, which was smaller than the total mean squared error (0.56) of the regression model that included the same 11 features identified through LASSO. There was a good overlap between the results obtained from LASSO and individual linear regression analyses as out of the 10 proteins revealed through LASSO, eight were identified through regression analyses (refer to Table 1).

### Pathway mapping and interaction network of proteins identified in the multianalyte panel

The proteins identified through LASSO were mapped to different molecular pathways such as amyloid fibre formation, lipoprotein assembly and clearance, innate immune system and transport of insulin-like growth factor. Further bioinformatic analyses revealed an interaction network among these proteins as shown in Additional file 1: Fig. S3. Similar protein-protein interactions were observed with the two tools. Of these 11 proteins (given that neuropilin-2 and cystatin-C are distinct proteins), eight were found to be interacting with one another. Despite having distinct biological functions, these proteins interacted through more than one pathway, suggesting an underlying physiological link.

## Discussion

The present study identified plasma proteins associated with overall cognitive function among ageing men without severe cognitive impairment. In age- and *APOE* ε4 carrier status-adjusted univariate analysis, 10 proteins, which are known to be associated with physiological processes such as lipid transport, immune responses, protection against oxidative damage and transmembrane transport, displayed an association with cognitive function. We suspected that the observed relationship between protein markers and cognition might be impacted by the presence of genetic variants linked to AD risk. Allelic variants belonging to four genes: *ABCA7*, *CLU*, *BDNF* and *MS4A6A*, showed an interaction with some of the protein markers for predicting cognitive function, suggesting that individuals carrying the risk alleles may show an altered biomarker profile. These interactions may further aid our understanding of the underlying biology of the disease. For instance, the protein apolipoprotein C-I, which plays a major role in lipid metabolism, displayed interaction with risk variants of *ABCA7* and *CLU* genes that also have a role in lipid metabolism. The gene *ABCA7* encodes for a transmembrane protein involved in the packaging of lipids into lipoprotein particles, while *CLU* encodes for an apolipoprotein that transports cholesterol in the brain [29, 30]. These interactions suggest a possible role for lipid transport and metabolism in modulating cognitive function. The effect of genotype on blood protein profiles for AD has been previously demonstrated by Soares et al. who found the *APOE* genotype to be associated with a unique biochemical plasma profile [3]. However, not much is known about the impact of other risk-conferring genetic variants on plasma biomarkers as *APOE* remains the most studied genetic risk factor to date even though AD can develop among individuals without the *APOE* ε4 risk allele.

Next, interactions were explored between protein markers and variables related to bone health and mood disorders. Poor bone health and mood disorders, especially depression, are some of the known modifiable risk factors associated with AD, and it is worthwhile to investigate whether their presence influences the relationship between biomarker levels and cognitive function. Bone mineral density at the femoral neck, total hip and spine along with a history of major osteoporotic fracture and diagnosis of a mood disorder, modified the relationship between plasma protein markers and cognition. Overall, the interaction results highlight the need to include genetic variants and other risk factors in biomarker studies, which face the inherent challenge of reproducibility. This is more important for health conditions such as AD, which is not driven by a single causative factor but arises due to a complex interplay between genetic and environmental risk factors. As the analyses above focused on a single protein analyte, a multianalyte panel associated with cognitive function was identified that comprised 10 protein markers and age. Eight of these plasma proteins were also found to be associated with cognitive function through individual regression analyses. Further analysis revealed dysregulation of pathways such as amyloid fibre formation and other biological mechanisms that may not directly relate to Aβ pathology such as lipoprotein assembly and immune responses. Encouragingly, proteins such as apolipoprotein A-I, apolipoprotein C-I and cystatin-C, which were identified through both univariate regression analyses and LASSO, are some of the most widely investigated plasma biomarkers for cognitive decline [31–34]. Among these proteins, the highest effect size was observed for apolipoprotein A-I through regression analyses wherein increasing levels of this protein were associated with better cognitive function. This is consistent with previous studies that have demonstrated that reduced blood apolipoprotein A-I levels are associated with an increased risk of cognitive decline [31, 35, 36].

Although these biomarker findings have the potential to be used for wide-scale population screening, they need to be first replicated across independent population cohorts to ensure high accuracy and reproducibility. Future prospective studies are required to see whether these protein markers can also predict any long-term cognitive change. Despite these challenges, blood-based screening to detect early cognitive changes and identify high-risk individuals offers several advantages over expensive PET imaging with limited availability or CSF measurements that involve an invasive lumbar puncture. Furthermore, high-throughput mass spectrometric platforms allow simultaneous quantitation of multiple proteins using sample volume as low as 30 μL, facilitating efficient blood-based screening tests [5].

This was a one-of-a-kind study that highlights the need to investigate the role of genetic risk factors other than *APOE* and health conditions in biomarker analysis. Our study was strengthened by the use of a population-based cohort where participants were drawn at random from the general population and did not comprise individuals with severe cognitive impairment or dementia. Also, an ethnically homogeneous population may yield more precise results. However, our findings may not be generalisable to other populations, and thus, future studies are required to extend these findings to other ethnically diverse cohorts. The present study included only male participants as cognitive function using CBB was evaluated for the first time for the GOS male cohort in their 15-year follow-up phase. We are collecting data for the female cohort in their current follow-up and plan to conduct a similar study for women in the future when their follow-up assessment phase is completed. Also, as this was an exploratory study, in order to mitigate the possibility of a false discovery rate, future confirmatory studies with adjustments for multiple comparisons are expected. Another limitation of our study was the lack of brain imaging data to correlate with biochemical findings.

Overall, this study supports the hypothesis that a relationship between plasma protein levels and cognitive function exists, and a blood-based proteomic signature can be exploited to enrich participants for more comprehensive AD testing. Early screening of high-risk groups may provide an opportunity to make lifestyle or pharmacological interventions before the onset of clinical symptoms, which become apparent only after irreversible neurological damage has occurred [2]. The study also underscores the importance of including information on genetic risk factors and other health conditions associated with AD in order to establish reproducible biomarkers.

### Supplementary Information

**Additional file 1:** **Fig. S1.** GOS participant selection flow diagram. **Fig. S2.** Features identified using LASSO regression for predicting cognitive function. **Fig. S3.** Interaction networks of proteins identified in the multianalyte panel. A Interaction networks generated using NetworkAnalyst. B Interaction networks generated using STRINGS. *APOA1 = Apolipoprotein A-I, CPN1 = Carboxypeptidase N catalytic chain, CST3 = Cystatin-C, ORM1 = Alpha-1-acid glycoprotein 1, AMBP = Alpha-1-microglobulin/bikunin precursor, APOC1 = Apolipoprotein C-I, LYZ = Lysozyme C and C1QB = Complement C1q subcomponent subunit B. **Table S1.** List of 269 proteins measured in blood plasma samples. **Table S2.** The distribution of risk alleles among the study participants.

## Data Availability

The datasets used and/or analysed during the current study are available from the senior authors upon reasonable request.
