# Supplementary material for: A plasma protein signature associated with cognitive function in men without severe cognitive impairment
Source: Alzheimers Res Ther. 2023 Sep 1;15:148. doi: 10.1186/s13195-023-01294-7 (PMC10472730; doi:10.1186/s13195-023-01294-7)
Supplement: Supplementary file 1 — Additional file 1: Fig. S1. GOS participant selection flow diagram. Fig. S2. Features identified using LASSO regression for predicting cognitive function. Fig. S3. Interaction networks of proteins identified in the multianalyte panel. A Interaction networks generated using NetworkAnalyst. B Interaction networks generated using STRINGS. *APOA1 = Apolipoprotein A-I, CPN1 = Carboxypeptidase N catalytic chain, CST3 = Cystatin-C, ORM1 = Alpha-1-acid glycoprotein 1, AMBP = Alpha-1-microglobulin/bikunin precursor, APOC1 = Apolipoprotein C-I, LYZ = Lysozyme C and C1QB = Complement C1q subcomponent subunit B. Table S1. List of 269 proteins measured in blood plasma samples. Table S2. The distribution of risk alleles among the study participants. [file 13195_2023_1294_MOESM1_ESM.docx]

**A plasma protein signature associated with cognitive function in men without severe cognitive impairment**

Kanika Mehta^1,2^, Mohammadreza Mohebbi^1,3^, Julie A. Pasco^1,4,5,6^, Lana J. Williams^1,6^, Sophia X. Sui^1^, Ken Walder^1^, Boon Lung Ng^7^, Veer Bala Gupta^1*^

^1^Deakin University, IMPACT – The Institute for Mental and Physical Health and Clinical Translation, School of Medicine, Geelong, VIC, Australia. ^2^Baker Heart and Diabetes Institute, Melbourne, Australia. ^3^Biostatistics Unit, Faculty of Health, Deakin University, Burwood, VIC, Australia. ^4^Department of Medicine-Western Health, The University of Melbourne, St Albans, VIC, Australia. ^5^Department of Epidemiology and Preventive Medicine, Monash University, Prahran, VIC, Australia. ^6^Barwon Health, Geelong, VIC, Australia. ^7^Department of Geriatric Medicine, Barwon Health, Geelong, VIC, Australia.

***Correspondence**

Veer Bala Gupta, Deakin University, IMPACT – The Institute for Mental and Physical Health and Clinical Translation, School of Medicine, Geelong, VIC 3216, Australia.

veer.gupta@deakin.edu.au

**Supplementary Fig. 1 GOS participant selection flow diagram.**

**
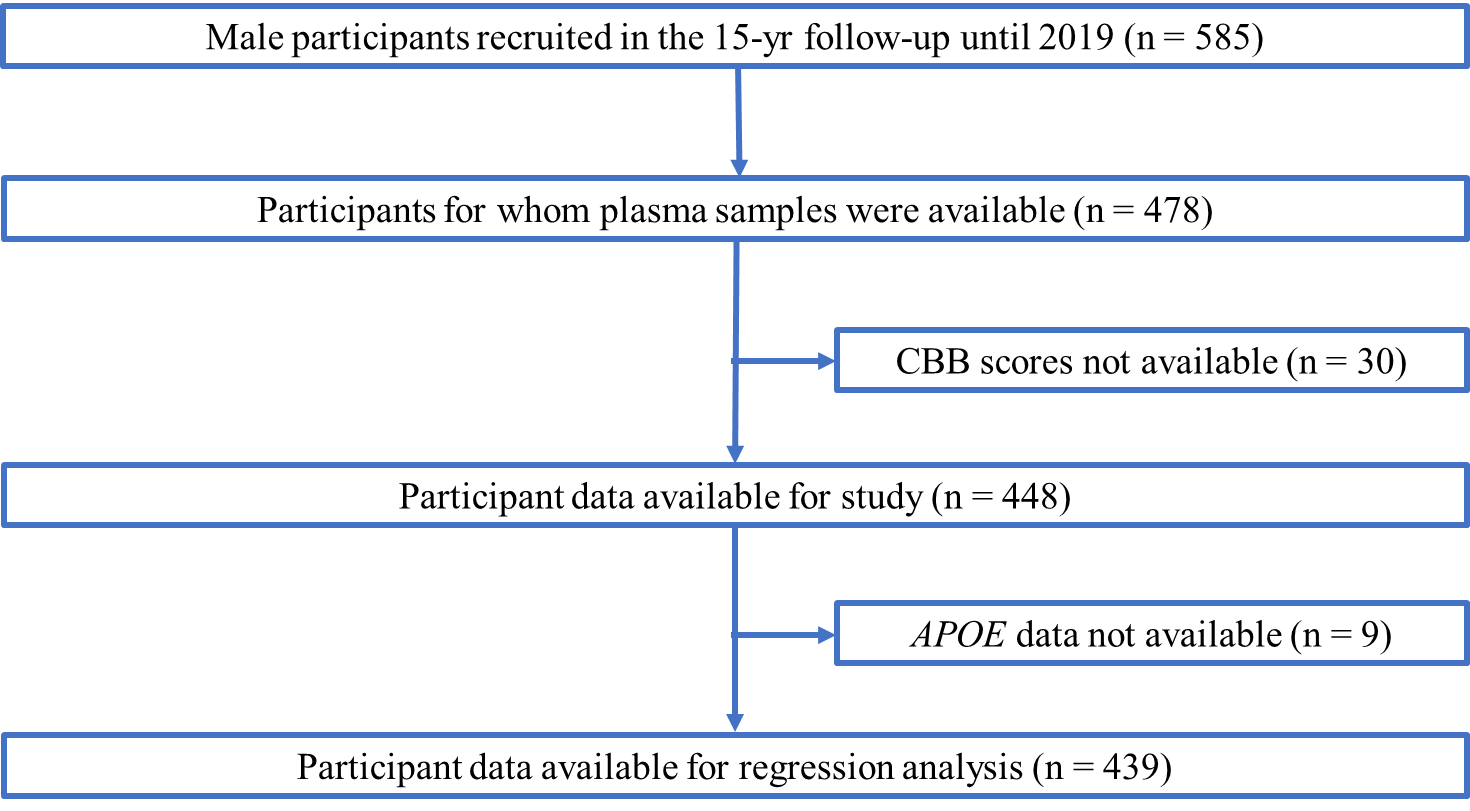
**

**Supplementary Fig. 2 Features identified using LASSO regression for predicting cognitive function.**

**Supplementary Fig. 3 Protein-protein interaction networks of proteins identified through the multianalyte panel. A** Interaction networks generated using NetworkAnalyst. **B** Interaction networks generated using STRINGS.

*APOA1 = Apolipoprotein A-I, CPN1 = Carboxypeptidase N catalytic chain, CST3 = Cystatin-C, ORM1 = Alpha-1-acid glycoprotein 1, AMBP = Alpha-1-microglobulin/bikunin precursor, APOC1 = Apolipoprotein C-I, LYZ = Lysozyme C and C1QB = Complement C1q subcomponent subunit B.


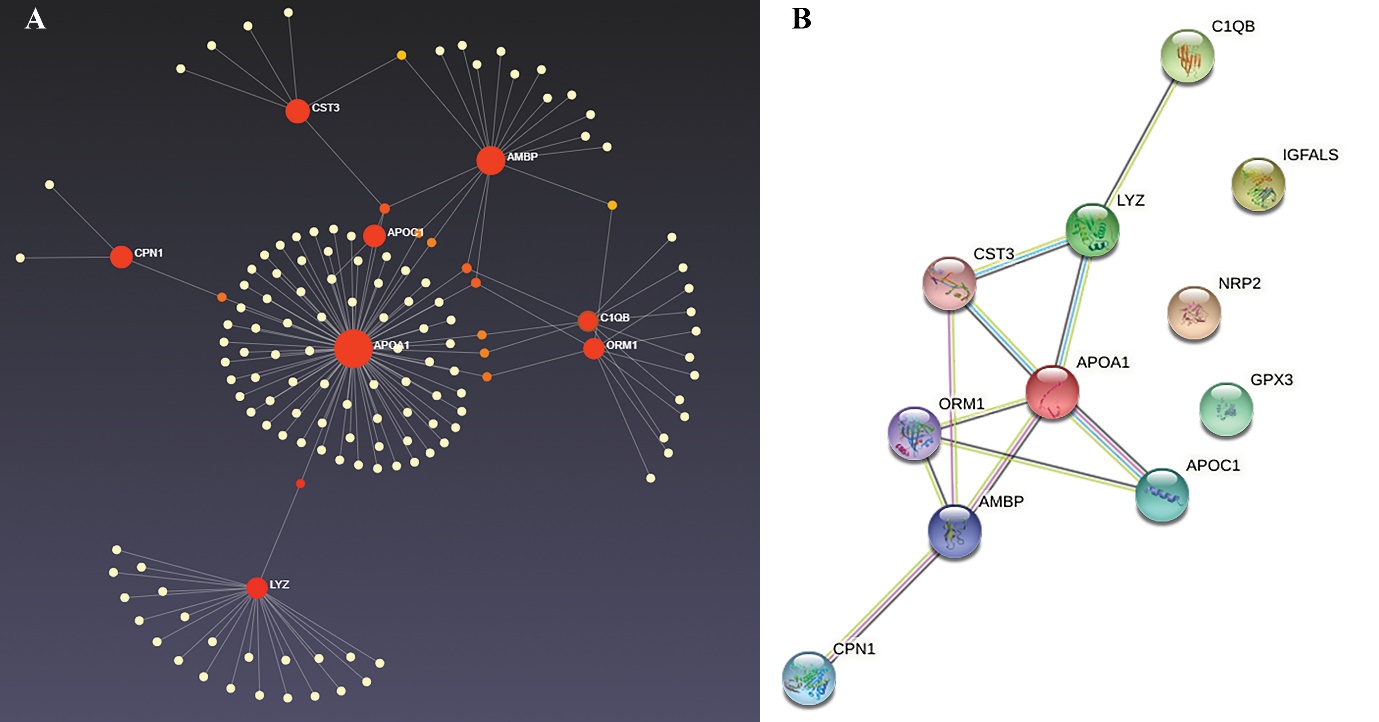


**Supplementary Table 1.** List of 269 protein markers measured in blood plasma samples.

| **Protein Name** | **Uniprot Accession No.** | **Peptide** |
| --- | --- | --- |
| 6 kDa heat shock protein, mitochondrial | P10809 | GIIDPTK |
| 72 kDa type IV collagenase | P08253 | IDAVYEAPQEEK |
| Endoplasmic reticulum chaperone BiP | P11021 | ITPSYVAFTPEGER |
| A disintegrin and metalloproteinase with thrombospondin motifs 2 | O95450 | IILLSYGK |
| A disintegrin and metalloproteinase with thrombospondin motifs 20 | P59510 | IPAGATNVDIR |
| A disintegrin and metalloproteinase with thrombospondin motifs 9 | Q9P2N4 | LYNPDVR |
| POTE ankyrin domain family member F, Actin, cytoplasmic 1, Actin, aortic smooth muscle, Actin, cytoplasmic 2, Actin, gamma-enteric smooth muscle, Actin, alpha cardiac muscle 1, Actin, alpha skeletal muscle, Beta-actin-like protein 2, POTE ankyrin domain family member E, Putative beta-actin-like protein 3 | A5A3E0, P60709, P62736, P63261, P63267, P68032, P68133, Q562R1, Q6S8J3, Q9BYX7 | SYELPDGQVITIGNER |
| Adhesion G protein-coupled receptor F5 | Q8IZF2 | DVIVHPLPLK |
| Adipocyte plasma membrane-associated protein | Q9HDC9 | LLEYDTVTR |
| Adiponectin | Q15848 | IFYNQQNHYDGSTGK |
| Pro-adrenomedullin | P35318 | LDVASEFR |
| Afamin | P43652 | DADPDTFFAK |
| Alpha-1-acid glycoprotein 1 | P02763 | NWGLSVYADKPETTK |
| Alpha-1-antichymotrypsin | P01011 | EIGELYLPK |
| Alpha-1-antitrypsin | P01009 | SVLGQLGITK |
| Alpha-1B-glycoprotein | P04217 | LETPDFQLFK |
| Alpha-2-antiplasmin | P08697 | LGNQEPGGQTALK |
| Alpha-2-HS-glycoprotein | P02765 | FSVVYAK |
| Alpha-2-macroglobulin | P01023 | AIGYLNTGYQR |
| Angiogenin | P03950 | DINTFIHGNK |
| Angiopoietin-related protein 3 | Q9Y5C1 | DLVFSTWDHK |
| Angiotensinogen | P01019 | ALQDQLVLVAAK |
| Antithrombin-III | P01008 | DDLYVSDAFHK |
| Apolipoprotein A-I | P02647 | ATEHLSTLSEK |
| Apolipoprotein A-II | P02652 | SPELQAEAK |
| Apolipoprotein A-IV | P06727 | LGEVNTYAGDLQK |
| Apolipoprotein B-1 | P04114 | FPEVDVLTK |
| Apolipoprotein C-I | P02654 | EWFSETFQK |
| Apolipoprotein C-II | P02655 | TYLPAVDEK |
| Apolipoprotein C-III | P02656 | GWVTDGFSSLK |
| Apolipoprotein C-IV | P55056 | ELLETVVNR |
| Apolipoprotein D | P05090 | NILTSNNIDVK |
| Apolipoprotein E | P02649 | LGPLVEQGR |
| Apolipoprotein F | Q13790 | SGVQQLIQYYQDQK |
| Apolipoprotein L1 | O14791 | VAQELEEK |
| Apolipoprotein M | O95445 | AFLLTPR |
| Apolipoprotein(a) | P08519 | GTYSTTVTGR |
| Aromatase | P11511 | NMLEMIFTPR |
| Atrial natriuretic peptide receptor 1 | P16066 | ITDYGLESFR |
| Attractin | O75882 | SVNNVVVR |
| Autism susceptibility gene 2 protein | Q8WXX7 | ALSLASSSGSDK |
| B-cell scaffold protein with ankyrin repeats | Q8NDB2 | LTIVHHPGGK |
| Beta-2-glycoprotein 1 | P02749 | ATVVYQGER |
| Beta-2-microglobulin | P61769 | VNHVTLSQPK |
| Beta-Ala-His dipeptidase | Q96KN2 | ALEQDLPVNIK |
| Beta-nerve growth factor | P01138 | TTATDIK |
| Biotinidase | P43251 | SHLIIAQVAK |
| C4b-binding protein alpha chain | P04003 | EDVYVVGTVLR |
| Cadherin-13 | P55290 | INENTGSVSVTR |
| Cadherin-5 | P33151 | ELDSTGTPTGK |
| Calcitonin gene-related peptide 1 | P06881 | NNFVPTNVGSK |
| Calcitonin | P01258 | FHTFPQTAIGVGAPGK |
| Calponin-1 | P51911 | VNVGVK |
| Carbonic anhydrase 1 | P00915 | VLDALQAIK |
| Carboxypeptidase B2 | Q96IY4 | IAWHVIR |
| Carboxypeptidase N catalytic chain | P15169 | SIPQVSPVR |
| Carboxypeptidase N subunit 2 | P22792 | AGGSWDLAVQER |
| Cartilage acidic protein 1 | Q9NQ79 | GVASLFAGR |
| Cathelicidin antimicrobial peptide | P49913 | AIDGINQR |
| Cation-independent mannose-6-phosphate receptor | P11717 | GHQAFDVGQPR |
| CD4 ligand | P29965 | SQFEGFVK |
| CD44 antigen | P16070 | YGFIEGHVVIPR |
| CD5 antigen-like | O43866 | LVGGLHR |
| Ceruloplasmin | P00450 | IYHSHIDAPK |
| Cholesteryl ester transfer protein | P11597 | GVSLFDIINPEIITR |
| Cholinesterase | P06276 | YLTLNTESTR |
| Chromogranin-A | P10645 | ELQDLALQGAK |
| Claudin-5 | O00501 | PDLSFPVK |
| Clusterin | P10909 | ELDESLQVAER |
| Coagulation factor IX | P00740 | SALVLQYLR |
| Coagulation factor V | P12259 | AEVDDVIQVR |
| Coagulation factor VII | P08709 | VSQYIEWLQK |
| Coagulation factor VIII | P00451 | LHPTHYSIR |
| Coagulation factor X | P00742 | MLEVPYVDR |
| Coagulation factor XI | P03951 | TSESGLPSTR |
| Coagulation factor XII | P00748 | EQPPSLTR |
| Coagulation factor XIII A chain | P00488 | GTYIPVPIVSELQSGK |
| Coagulation factor XIII B chain | P05160 | IQTHSTTYR |
| Collagen alpha-1(I) chain | P02452 | GVVGLPGQR |
| Collagen alpha-1(III) chain | P02461 | GGAGPPGPEGGK |
| Collagen alpha-1(XVIII) chain | P39060 | AVGLAGTFR |
| Collagen alpha-2(I) chain | P08123 | GVVGPQGAR |
| Complement C1q subcomponent subunit A | P02745 | PAFSAIR |
| Complement C1q subcomponent subunit B | P02746 | IAFSATR |
| Complement C1q subcomponent subunit C | P02747 | FQSVFTVTR |
| Complement C1r subcomponent | P00736 | GLTLHLK |
| Complement C1r subcomponent-like protein | Q9NZP8 | VVVHPDYR |
| Complement C1s subcomponent | P09871 | TNFDNDIALVR |
| Complement C2 | P06681 | HAFILQDTK |
| Complement C3 | P01024 | TGLQEVEVK |
| Complement C5 | P01031 | VFQFLEK |
| Complement component C6 | P13671 | DLHLSDVFLK |
| Complement component C7 | P10643 | AASGTQNNVLR |
| Complement component C8 alpha chain | P07357 | MESLGITSR |
| Complement component C8 beta chain | P07358 | SDLEVAHYK |
| Complement component C9 | P02748 | LSPIYNLVPVK |
| Complement factor B | P00751 | EELLPAQDIK |
| Complement factor D | P00746 | THHDGAITER |
| Complement factor H | P08603 | SSQESYAHGTK |
| Complement factor I | P05156 | VFSLQWGEVK |
| Complement C4-A, Complement C4-B | P0C0L4, P0C0L5 | VGDTLNLNLR |
| Complement C4-A, Complement C4-B | P0C0L4, P0C0L5 | VLSLAQEQVGGSPEK |
| Corticosteroid-binding globulin | P08185 | WSAGLTSSQVDLYIPK |
| Creatine kinase B-type | P12277 | DLFDPIIEDR |
| C-reactive protein | P02741 | AFVFPK |
| Creatine kinase M-type | P06732 | FEEILTR |
| Cystatin-C | P01034 | ALDFAVGEYNK |
| Desmoplakin | P15924 | AELIVQPELK |
| Dickkopf-related protein 1, Dickkopf-related protein 2 | O94907, Q9UBU2 | GSHGLEIFQR |
| Di-N-acetylchitobiase | Q01459 | ATYIQNYR |
| Elastin | P15502 | LPGGYGLPYTTGK |
| Endothelial lipase | Q9Y5X9 | LVSALHTR |
| Endothelial protein C receptor | Q9UNN8 | TLAFPLTIR |
| Epidermal growth factor receptor | P00533 | IPLENLQIIR |
| E-selectin | P16581 | YTHLVAIQNK |
| Extracellular matrix protein 1 | Q16610 | NVALVSGDTENAK |
| Fatty acid-binding protein, heart | P05413 | SLGVGFATR |
| Ferritin heavy chain | P02794 | NVNQSLLELHK |
| Ferritin light chain | P02792 | LGGPEAGLGEYLFER |
| Fetuin-B | Q9UGM5 | LVVLPFPK |
| Fibrinogen alpha chain | P02671 | VQHIQLLQK |
| Fibrinogen beta chain | P02675 | HQLYIDETVNSNIPTNLR |
| Fibrinogen gamma chain | P02679 | YEASILTHDSSIR |
| Fibronectin | P02751 | HTSVQTTSSGSGPFTDVR |
| Fibulin-1 | P23142 | TGYYFDGISR |
| Ficolin-2 | Q15485 | GTHGSFANGINWK |
| Ficolin-3 | O75636 | YAVSEAAAHK |
| Follistatin-related protein 1, Early endosome antigen 1 | Q12841, Q15075 | YVQELQK |
| Fructose-bisphosphate aldolase B | P05062 | ALQASALAAWGGK |
| Galectin-3 | P17931 | IALDFQR |
| Galectin-3-binding protein | Q08380 | SDLAVPSELALLK |
| Gamma-enolase | P09104 | YITGDQLGALYQDFVR |
| Gelsolin | P06396 | AGALNSNDAFVLK |
| Glial fibrillary acidic protein | P14136 | LADVYQAELR |
| Glutamate receptor ionotropic, NMDA 2A | Q12879 | FSYIPEAK |
| Glutamate receptor ionotropic, NMDA 2B | Q13224 | EPGGPSFTIGK |
| Glutathione peroxidase 3 | P22352 | QEPGENSEILPTLK |
| Haptoglobin, Haptoglobin-related protein | P00738, P00739 | DIAPTLTLYVGK |
| Glutathione S-transferase P | P09211 | TLGLYGK |
| Heat shock protein beta-1 | P04792 | LFDQAFGLPR |
| Hemopexin | P02790 | NFPSPVDAAFR |
| Heparin cofactor 2 | P05546 | TLEAQLTPR |
| Hepatocyte growth factor-like protein | P26927 | SPLNDFQVLR |
| Histidine-rich glycoprotein | P04196 | ADLFYDVEALDLESPK |
| Hornerin | Q86YZ3 | GSGSGQSPSSGQHGTGFGR |
| Hyaluronan-binding protein 2 | Q14520 | VVLGDQDLK |
| Immunoglobulin heavy constant gamma 1, Immunoglobulin gamma-1 heavy chain | P01857, P0DOX5 | GPSVFPLAPSSK |
| Immunoglobulin heavy constant mu, Immunoglobulin mu heavy chain | P01871, P0DOX6 | VSVFVPPR |
| IgGFc-binding protein | Q9Y6R7 | GATTSPGVYELSSR |
| Hemoglobin subunit alpha | P69905 | VGAHAGEYGAEALER |
| Immunoglobulin kappa variable 4-1 | P06312 | NYLAWYQQKPGQPPK |
| Insulin-like growth factor I | P05019 | GFYFNKPTGYGSSSR |
| Insulin-like growth factor-binding protein 1 | P08833 | ALPGEQQPLHALTR |
| Insulin-like growth factor-binding protein 2 | P18065 | LIQGAPTIR |
| Insulin-like growth factor-binding protein 3 | P17936 | FLNVLSPR |
| Insulin-like growth factor-binding protein complex acid labile subunit | P35858 | NLIAAVAPGAFLGLK |
| Inter-alpha-trypsin inhibitor heavy chain H1 | P19827 | GSLVQASEANLQAAQDFVR |
| Inter-alpha-trypsin inhibitor heavy chain H2 | P19823 | SLAPTAAAK |
| Inter-alpha-trypsin inhibitor heavy chain H4 | Q14624 | SPEQQETVLDGNLIIR |
| Intercellular adhesion molecule 1 | P05362 | LLGIETPLPK |
| Interleukin-1 | P22301 | AHVNSLGENLK |
| Interleukin-6 | P05231 | FESSEEQAR |
| Interstitial collagenase | P03956 | AFQLWSNVTPLTFTK |
| Kallistatin | P29622 | VGSALFLSHNLK |
| Keratin, type I cytoskeletal 1 | P13645 | SLLEGEGSSGGGGR |
| Keratin, type I cytoskeletal 9 | P35527 | TLLDIDNTR |
| Keratin, type II cytoskeletal 2 epidermal | P35908 | YEELQVTVGR |
| Kininogen-1 | P01042 | DIPTNSPELEETLTHTITK |
| Lactotransferrin | P02788 | YLGPQYVAGITNLK |
| Leucine-rich alpha-2-glycoprotein | P02750 | DLLLPQPDLR |
| Lipopolysaccharide-binding protein | P18428 | ITLPDFTGDLR |
| L-selectin | P14151 | AEIEYLEK |
| Lumican | P51884 | SLEDLQLTHNK |
| Lysozyme C | P61626 | AWVAWR |
| Mannan-binding lectin serine protease 1 | P48740 | TGVITSPDFPNPYPK |
| Mannan-binding lectin serine protease 2 | O00187 | WPEPVFGR |
| Mannose-binding protein C | P11226 | WLTFSLGK |
| Matrix Gla protein | P08493 | NANTFISPQQR |
| Matrix metalloproteinase-9 | P14780 | AVIDDAFAR |
| Melanotransferrin | P08582 | YYDYSGAFR |
| Metalloproteinase inhibitor 1 | P01033 | GFQALGDAADIR |
| Metalloproteinase inhibitor 2 | P16035 | EYLIAGK |
| Metalloproteinase inhibitor 4 | Q99727 | VVPASADPADTEK |
| Microtubule-associated protein tau | P10636 | EADLPEPSEK |
| Mucin-16 | Q8WXI7 | ELGPYTLDR |
| Myelin basic protein | P02686 | GVDAQGTLSK |
| Myeloblastin | P24158 | LVNVVLGAHNVR |
| Myeloperoxidase | P05164 | VFFASWR |
| N(G),N(G)-dimethylarginine dimethylaminohydrolase 1 | O94760 | TPEEYPESAK |
| N-acetylmuramoyl-L-alanine amidase | Q96PD5 | AGLLRPDYALLGHR |
| Natriuretic peptides B | P16860 | EVATEGIR |
| Neuropilin-2, Cystatin-C | O60462, P01034 | ALQVVR |
| Neutrophil gelatinase-associated lipocalin | P80188 | ITLYGR |
| Nucleoside diphosphate kinase A | P15531 | PFFAGLVK |
| Occludin | Q16625 | SLQSELDEINK |
| Osteopontin | P10451 | GDSVVYGLR |
| Oxidized low-density lipoprotein receptor 1 | P78380 | LEGQISAR |
| Pappalysin-1 | Q13219 | AYLDVNELK |
| Peroxiredoxin-1 | Q06830 | ADEGISFR |
| Peroxiredoxin-2 | P32119 | GLFIIDGK |
| Phosphatidylcholine-sterol acyltransferase | P04180 | SSGLVSNAPGVQIR |
| Phosphatidylinositol-glycan-specific phospholipase D | P80108 | FGSSLITVR |
| Phospholipid transfer protein | P55058 | AVEPQLQEEER |
| Pigment epithelium-derived factor | P36955 | LQSLFDSPDFSK |
| Plasma protease C1 inhibitor | P05155 | FQPTLLTLPR |
| Plasma serine protease inhibitor | P05154 | GFQQLLQELNQPR |
| Plasminogen activator inhibitor 1 | P05121 | VFQQVAQASK |
| Plasminogen | P00747 | EAQLPVIENK |
| Plastin-2 | P13796 | ISFDEFIK |
| Platelet endothelial cell adhesion molecule | P16284 | SELVTVTESFSTPK |
| Platelet glycoprotein VI | Q9HCN6 | EGDPAPYK |
| Platelet-activating factor acetylhydrolase | Q13093 | GSVHQNFADFTFATGK |
| Pregnancy zone protein | P20742 | ISEITNIVSK |
| Proenkephalin-A | P01210 | ELLETGDNR |
| Prolactin | P01236 | IDNYLK |
| Protein AMBP | P02760 | HHGPTITAK |
| Protein S100-A12 | P80511 | GHFDTLSK |
| Protein S1-A9 | P06702 | DLQNFLK |
| Protein S1-B | P04271 | EQEVVDK |
| Protein Z-dependent protease inhibitor | Q9UK55 | ETSNFGFSLLR |
| Parkinson disease protein 7 | Q99497 | ALVILAK |
| Proteoglycan 4 | Q92954 | DQYYNIDVPSR |
| Prothrombin | P00734 | ELLESYIDGR |
| P-selectin | P16109 | TWTWVGTK |
| Ras GTPase-activating protein nGAP | Q9UJF2 | ETQSTPQSAPQVR |
| Resistin | Q9HD89 | IQEVAGSLIFR |
| Retinol-binding protein 4 | P02753 | YWGVASFLQK |
| Serotransferrin | P02787 | DGAGDVAFVK |
| Albumin | P02768 | LVNEVTEFAK |
| Serum amyloid A-1 protein, Serum amyloid A-2 protein | P0DJI8, P0DJI9 | EANYIGSDK |
| Serum amyloid A-4 protein | P35542 | GNYDAAQR |
| Serum amyloid P-component | P02743 | IVLGQEQDSYGGK |
| Serum paraoxonase/arylesterase 1 | P27169 | IFFYDSENPPASEVLR |
| Serum paraoxonase/lactonase 3 | Q15166 | ILIGTVFHK |
| Sex hormone-binding globulin | P04278 | TSSSFEVR |
| SPARC | P09486 | LEAGDHPVELLAR |
| Spermine oxidase | Q9NWM0 | YYSTTHGALLSGQR |
| Sterile alpha motif domain-containing protein 9-like | Q8IVG5 | ENVLDEVANAK |
| Stromelysin-1 | P08254 | TYFFVEDK |
| Target of Nesh-SH3 | Q7Z7G0 | IYLSDSLTGK |
| TBC1 domain family member 10A | Q9BXI6 | YLPGYYSEK |
| Tenascin | P24821 | FTTDLDSPR |
| Tenascin-X, Putative tenascin-XA | P22105, Q16473 | ILISGLEPSTPYR |
| Tetranectin | P05452 | NWETEITAQPDGGK |
| Thrombomodulin | P07204 | SSVAADVISLLLNGDGGVGR |
| Thrombospondin-1 | P07996 | GTLLALER |
| Thrombospondin-4 | P35443 | KPQDFLEELK |
| Thyroglobulin | P01266 | FSPDDSAGASALLR |
| Thyroxine-binding globulin | P05543 | AVLHIGEK |
| Tissue factor pathway inhibitor | P10646 | FYYNSVIGK |
| Tissue-type plasminogen activator | P00750 | VVPGEEEQK |
| Transcription factor SOX-11 | P35716 | AAQSGDYGGAGDDYVLGSLR |
| Transferrin receptor protein 1 | P02786 | GFVEPDHYVVVGAQR |
| Transthyretin | P02766 | GSPAINVAVHVFR |
| Tumor necrosis factor receptor superfamily member 1A | P19438 | LGLSDHEIDR |
| Tumor necrosis factor receptor superfamily member 1B | P20333 | DEQVPFSK |
| Vascular cell adhesion protein 1 | P19320 | NTVISVNPSTK |
| Vascular endothelial growth factor B | P49765 | VVSWIDVYTR |
| Vascular endothelial growth factor D | O43915 | DLIQHPK |
| Vascular non-inflammatory molecule 3 | Q9NY84 | TETPVSK |
| Vasorin | Q6EMK4 | YLQGSSVQLR |
| Vitamin D-binding protein | P02774 | VLEPTLK |
| Vitamin K-dependent protein C | P04070 | LGEYDLR |
| Vitamin K-dependent protein S | P07225 | VYFAGFPR |
| Vitamin K-dependent protein Z | P22891 | GLLSGWAR |
| Vitronectin | P04004 | FEDGVLDPDYPR |
| von Willebrand factor | P04275 | ILAGPAGDSNVVK |
| Xaa-Pro dipeptidase | P12955 | AVYEAVLR |
| Zinc-alpha-2-glycoprotein | P25311 | EIPAWVPFDPAAQITK |

**Supplementary Table 2.** The distribution of risk alleles among the study participants.

| **Participant ID** | ***CLU*_carrier** | ***PICALM*_carrier** | ***MS4A6A*_carrier** | ***BIN1*_carrier** | ***ABCA7*_carrier** | ***CR1*_carrier** | ***CD33*_carrier** | ***BDNF*_carrier** | ***CD2AP*_carrier** | ***APOE* ε4_carrier** |
| --- | --- | --- | --- | --- | --- | --- | --- | --- | --- | --- |
| 1 | 1 | 1 | 0 | 1 | 0 | 0 | 1 | 1 | 0 | 0 |
| 3 | 1 | 1 | 1 | 1 | 1 | 0 | 0 | 1 | 1 | 0 |
| 4 | 0 | 1 | 1 | 0 | 0 | 0 | 0 | 0 | 0 | 1 |
| 5 | 1 | 0 | 0 | 1 | 0 | 0 | 0 | 0 | 0 | 1 |
| 7 | 1 | 0 | 0 | 0 | 0 | 0 | 1 | 0 | 1 | 0 |
| 8 | 1 | 1 | 1 | 1 | 0 | 0 | 0 | 0 | 0 | 0 |
| 9 | 0 | 1 | 1 | 0 | 0 | 0 | 1 | 1 | 1 | 1 |
| 10 | 1 | 0 | 0 | 1 | 0 | 0 | 1 | 1 | 1 | 0 |
| 11 | 1 | 1 | 0 | 1 | 0 | 0 | 0 | 0 | 1 | 1 |
| 12 | 0 | 0 | 1 | 0 | 0 | 0 | 1 | 0 | 0 | 0 |
| 13 | 0 | 1 | 0 | 1 | 0 | 0 | 1 | 1 | 1 | 1 |
| 14 | 1 | 1 | 0 | 1 | 0 | 1 | 0 | 0 | 0 | 0 |
| 16 | 1 | 0 | 1 | 1 | 0 | 0 | 0 | 0 | 1 | 1 |
| 17 | 1 | 0 | 1 | 1 | 0 | 0 | 1 | 0 | 0 | 0 |
| 18 | 1 | 1 | 0 | 1 | 0 | 0 | 1 | 0 | 1 | 0 |
| 19 | 1 | 1 | 1 | 1 | 1 | 0 | 1 | 1 | 0 | 0 |
| 21 | 0 | 0 | 1 | 0 | 0 | 1 | 1 | 0 | 0 | 0 |
| 22 | 1 | 0 | 1 | 0 | 0 | 0 | 1 | 1 | 1 | 1 |
| 23 | 1 | 0 | 1 | 0 | 0 | 0 | 1 | 1 | 0 | 0 |
| 24 | 1 | 1 | 1 | 0 | 0 | 0 | 1 | 0 | 1 | 0 |
| 25 | 0 | 0 | 0 | 1 | 0 | 0 | 1 | 0 | 1 | 1 |
| 26 | 1 | 0 | 1 | 0 | 0 | 0 | 0 | 1 | 0 | 1 |
| 27 | 1 | 0 | 0 | 1 | 0 | 0 | 1 | 1 | 1 | 0 |
| 28 | 1 | 0 | 0 | 1 | 1 | 0 | 0 | 1 | 0 | 0 |
| 29 | 1 | 1 | 1 | 1 | 0 | 0 | 0 | 0 | 0 | 0 |
| 30 | 0 | 0 | 1 | 0 | 0 | 0 | 0 | 0 | 0 | 0 |
| 31 | 1 | 1 | 0 | 0 | 0 | 0 | 0 | 0 | 0 | 1 |
| 32 | 0 | 1 | 1 | 0 | 0 | 0 | 0 | 0 | 0 | 0 |
| 33 | 1 | 0 | 0 | 1 | 0 | 1 | 0 | 0 | 0 | 1 |
| 34 | 0 | 1 | 1 | 0 | 1 | 0 | 1 | 0 | 1 | 0 |
| 36 | 0 | 1 | 0 | 1 | 0 | 0 | 0 | 0 | 0 | 0 |
| 37 | 0 | 1 | 0 | 1 | 0 | 0 | 1 | 0 | 0 | 0 |
| 38 | 0 | 0 | 0 | 0 | 0 | 0 | 1 | 1 | 0 | 1 |
| 39 | 1 | 0 | 1 | 0 | 0 | 0 | 0 | 0 | 1 | 0 |
| 40 | 0 | 1 | 1 | 0 | 0 | 0 | 1 | 1 | 0 | 0 |
| 41 | 1 | 0 | 0 | 0 | 0 | 1 | 1 | 0 | 1 | 0 |
| 42 | 1 | 1 | 1 | 0 | 0 | 0 | 1 | 0 | 0 | 0 |
| 44 | 1 | 0 | 1 | 0 | 0 | 1 | 1 | 1 | 1 | 0 |
| 45 | 1 | 1 | 1 | 1 | 1 | 0 | 1 | 0 | 0 | 0 |
| 46 | 1 | 1 | 1 | 0 | 0 | 0 | 0 | 1 | 1 | 0 |
| 47 | 1 | 1 | 0 | 1 | 1 | 0 | 0 | 0 | 0 | 0 |
| 48 | 1 | 1 | 0 | 0 | 0 | 0 | 1 | 1 | 0 | 1 |
| 49 | 1 | 0 | 1 | 1 | 0 | 1 | 1 | 1 | 0 | 0 |
| 50 | 1 | 0 | 1 | 0 | 0 | 0 | 1 | 0 | 0 | 0 |
| 51 | 0 | 1 | 1 | 1 | 0 | 0 | 0 | 0 | 0 | 0 |
| 52 | 0 | 1 | 1 | 1 | 0 | 1 | 1 | 1 | 0 | 0 |
| 53 | 1 | 1 | 0 | 1 | 0 | 0 | 0 | 0 | 1 | 1 |
| 54 | 0 | 1 | 1 | 1 | 0 | 0 | 1 | 0 | 0 | 0 |
| 55 | 1 | 1 | 1 | 0 | 0 | 1 | 0 | 0 | 1 | 0 |
| 56 | 0 | 0 | 0 | 1 | 0 | 1 | 1 | 1 | 1 | 0 |
| 57 | 0 | 0 | 1 | 1 | 0 | 1 | 0 | 0 | 1 | 0 |
| 58 | 0 | 0 | 0 | 1 | 0 | 1 | 1 | 0 | 1 | 1 |
| 59 | 1 | 1 | 0 | 1 | 0 | 0 | 0 | 1 | 0 | 0 |
| 60 | 0 | 0 | 1 | 0 | 0 | 0 | 0 | 1 | 0 | 0 |
| 61 | 0 | 1 | 1 | 0 | 1 | 1 | 0 | 1 | 1 | 0 |
| 62 | 1 | 1 | 1 | 0 | 0 | 0 | 0 | 1 | 0 | 0 |
| 63 | 1 | 1 | 0 | 0 | 0 | 1 | 0 | 1 | 1 | 0 |
| 64 | 0 | 0 | 1 | 1 | 1 | 0 | 1 | 0 | 1 | 0 |
| 65 | 1 | 1 | 1 | 0 | 0 | 0 | 1 | 0 | 0 | 0 |
| 67 | 1 | 1 | 1 | 1 | 0 | 0 | 0 | 1 | 1 | 0 |
| 69 | 0 | 1 | 0 | 1 | 0 | 1 | 0 | 0 | 1 | 0 |
| 70 | 1 | 1 | 1 | 1 | 0 | 0 | 0 | 0 | 1 | 0 |
| 71 | 0 | 0 | 1 | 1 | 0 | 1 | 1 | 1 | 0 | 0 |
| 72 | 1 | 1 | 0 | 1 | 1 | 0 | 0 | 0 | 0 | 1 |
| 73 | 1 | 0 | 1 | 0 | 0 | 0 | 1 | 0 | 0 | 1 |
| 74 | 1 | 0 | 1 | 0 | 0 | 1 | 0 | 1 | 0 | 1 |
| 75 | 0 | 1 | 1 | 0 | 0 | 1 | 0 | 0 | 1 | 0 |
| 76 | 1 | 0 | 1 | 1 | 0 | 1 | 1 | 0 | 0 | 0 |
| 77 | 1 | 0 | 1 | 1 | 0 | 0 | 1 | 0 | 1 | 1 |
| 78 | 0 | 0 | 1 | 1 | 0 | 1 | 1 | 0 | 0 | 0 |
| 79 | 1 | 1 | 1 | 0 | 0 | 0 | 0 | 0 | 1 | 0 |
| 80 | 1 | 0 | 1 | 1 | 1 | 0 | 0 | 0 | 0 | 0 |
| 82 | 1 | 1 | 1 | 1 | 0 | 0 | 1 | 0 | 1 | 0 |
| 83 | 1 | 1 | 0 | 0 | 0 | 0 | 1 | 0 | 0 | 1 |
| 84 | 0 | 0 | 1 | 1 | 0 | 1 | 1 | 0 | 0 | 1 |
| 85 | 1 | 0 | 0 | 1 | 0 | 1 | 0 | 0 | 1 | 1 |
| 86 | 0 | 1 | 1 | 1 | 0 | 0 | 1 | 0 | 0 | 0 |
| 87 | 1 | 0 | 0 | 0 | 0 | 0 | 1 | 0 | 0 | 0 |
| 88 | 1 | 1 | 1 | 0 | 1 | 0 | 1 | 1 | 1 | 1 |
| 89 | 0 | 1 | 1 | 0 | 1 | 1 | 1 | 0 | 0 | 1 |
| 90 | 0 | 1 | 1 | 1 | 0 | 1 | 1 | 1 | 1 | 1 |
| 91 | 1 | 1 | 1 | 1 | 0 | 0 | 1 | 1 | 0 | 1 |
| 92 | 0 | 1 | 1 | 1 | 1 | 0 | 0 | 0 | 0 | 0 |
| 93 | 1 | 0 | 1 | 0 | 1 | 0 | 0 | 1 | 0 | 0 |
| 94 | 1 | 0 | 1 | 0 | 1 | 1 | 0 | 0 | 0 | 0 |
| 95 | 1 | 1 | 1 | 1 | 0 | 0 | 0 | 1 | 0 | 0 |
| 96 | 1 | 0 | 0 | 0 | 0 | 0 | 0 | 0 | 0 | 0 |
| 97 | 1 | 1 | 0 | 1 | 0 | 0 | 1 | 1 | 1 | 0 |
| 98 | 0 | 1 | 1 | 1 | 1 | 0 | 1 | 0 | 1 | 0 |
| 100 | 1 | 1 | 1 | 0 | 0 | 0 | 1 | 1 | 1 | 0 |
| 101 | 1 | 0 | 0 | 1 | 1 | 0 | 1 | 0 | 0 | 0 |
| 103 | 1 | 0 | 1 | 0 | 0 | 0 | 1 | 1 | 1 | 0 |
| 104 | 1 | 1 | 1 | 0 | 0 | 0 | 1 | 0 | 1 | 0 |
| 105 | 1 | 0 | 1 | 1 | 1 | 1 | 0 | 0 | 1 | 0 |
| 106 | 1 | 1 | 1 | 1 | 0 | 0 | 1 | 0 | 1 | 0 |
| 107 | 1 | 0 | 1 | 0 | 0 | 1 | 1 | 1 | 1 | 0 |
| 108 | 1 | 0 | 0 | 0 | 0 | 1 | 0 | 0 | 0 | 0 |
| 109 | 0 | 0 | 1 | 1 | 0 | 1 | 1 | 0 | 0 | 0 |
| 110 | 1 | 1 | 0 | 1 | 0 | 1 | 1 | 0 | 1 | 0 |
| 112 | 0 | 1 | 1 | 0 | 0 | 0 | 1 | 1 | 0 | 0 |
| 113 | 0 | 1 | 0 | 1 | 1 | 0 | 0 | 0 | 1 | 0 |
| 114 | 1 | 1 | 0 | 1 | 0 | 1 | 1 | 0 | 0 | 0 |
| 115 | 1 | 1 | 0 | 1 | 0 | 0 | 1 | 1 | 1 | 1 |
| 116 | 0 | 0 | 1 | 1 | 0 | 1 | 0 | 0 | 1 | 0 |
| 117 | 1 | 1 | 1 | 0 | 0 | 0 | 1 | 1 | 1 | 0 |
| 118 | 1 | 0 | 1 | 0 | 1 | 1 | 0 | 0 | 0 | 0 |
| 119 | 1 | 0 | 1 | 0 | 0 | 0 | 1 | 0 | 1 | 0 |
| 120 | 1 | 0 | 1 | 0 | 1 | 0 | 1 | 1 | 0 | 0 |
| 121 | 0 | 1 | 1 | 0 | 0 | 0 | 1 | 1 | 0 | 0 |
| 123 | 0 | 0 | 1 | 1 | 1 | 0 | 1 | 1 | 1 | 0 |
| 124 | 1 | 1 | 0 | 0 | 0 | 1 | 0 | 0 | 1 | 0 |
| 125 | 0 | 1 | 1 | 1 | 0 | 0 | 1 | 1 | 0 | 0 |
| 126 | 1 | 0 | 0 | 0 | 0 | 1 | 0 | 0 | 0 | 1 |
| 127 | 1 | 0 | 1 | 0 | 0 | 1 | 1 | 0 | 1 | 0 |
| 128 | 1 | 1 | 1 | 0 | 0 | 0 | 0 | 0 | 1 | 0 |
| 129 | 1 | 1 | 0 | 0 | 0 | 0 | 1 | 1 | 1 | 0 |
| 130 | 1 | 0 | 1 | 1 | 1 | 0 | 0 | 0 | 1 | 0 |
| 132 | 1 | 1 | 1 | 1 | 1 | 0 | 0 | 0 | 1 | 1 |
| 133 | 1 | 1 | 0 | 1 | 0 | 1 | 0 | 1 | 0 | 1 |
| 134 | 1 | 0 | 1 | 0 | 0 | 1 | 0 | 0 | 0 | 0 |
| 135 | 0 | 1 | 1 | 1 | 0 | 0 | 0 | 1 | 0 | 1 |
| 136 | 1 | 1 | 1 | 0 | 0 | 1 | 1 | 0 | 1 | 1 |
| 137 | 1 | 0 | 0 | 1 | 0 | 1 | 0 | 0 | 1 | 0 |
| 138 | 1 | 0 | 1 | 0 | 0 | 0 | 1 | 0 | 1 | 0 |
| 139 | 1 | 1 | 1 | 1 | 0 | 0 | 1 | 0 | 0 | 0 |
| 140 | 1 | 1 | 0 | 1 | 0 | 1 | 0 | 1 | 0 | 0 |
| 141 | 0 | 1 | 0 | 0 | 1 | 1 | 1 | 0 | 0 | 0 |
| 142 | 1 | 0 | 0 | 1 | 0 | 0 | 1 | 1 | 0 | 0 |
| 143 | 1 | 0 | 1 | 0 | 0 | 0 | 0 | 0 | 1 | 1 |
| 144 | 0 | 0 | 1 | 1 | 0 | 0 | 1 | 0 | 0 | 0 |
| 145 | 1 | 1 | 1 | 0 | 0 | 0 | 1 | 0 | 1 | 1 |
| 146 | 1 | 1 | 1 | 1 | 0 | 0 | 0 | 1 | 1 | 1 |
| 147 | 1 | 1 | 1 | 1 | 0 | 0 | 0 | 0 | 1 | 0 |
| 148 | 0 | 1 | 1 | 1 | 0 | 0 | 0 | 1 | 1 | 0 |
| 149 | 0 | 1 | 1 | 0 | 0 | 0 | 0 | 0 | 1 | 1 |
| 150 | 1 | 1 | 1 | 0 | 0 | 0 | 0 | 1 | 0 | 0 |
| 151 | 0 | 0 | 1 | 1 | 0 | 1 | 1 | 0 | 1 | 0 |
| 152 | 0 | 1 | 1 | 0 | 0 | 0 | 0 | 0 | 1 | 1 |
| 153 | 1 | 1 | 0 | 1 | 0 | 0 | 1 | 1 | 0 | 0 |
| 154 | 1 | 0 | 1 | 1 | 0 | 0 | 1 | 0 | 0 | 0 |
| 155 | 0 | 0 | 0 | 1 | 0 | 0 | 0 | 1 | 1 | 0 |
| 156 | 0 | 1 | 0 | 1 | 0 | 0 | 1 | 0 | 1 | 0 |
| 157 |  | 1 | 1 | 0 | 0 | 1 | 1 | 0 | 1 | 0 |
| 158 | 1 | 0 | 1 | 0 | 0 | 0 | 1 | 1 | 1 | 0 |
| 159 | 1 | 0 | 1 | 1 | 0 | 0 | 1 | 0 | 1 | 0 |
| 160 | 1 | 1 | 1 | 0 | 1 | 0 | 1 | 0 | 1 | 0 |
| 161 | 0 | 1 | 1 | 1 | 0 | 0 | 0 | 0 | 0 | 0 |
| 162 | 0 | 1 | 0 | 1 | 0 | 0 | 0 | 1 | 1 | 0 |
| 163 | 0 | 1 | 1 | 0 | 0 | 0 | 1 | 0 | 1 | 0 |
| 164 | 0 | 0 | 0 | 0 | 0 | 1 | 0 | 1 | 0 | 0 |
| 165 | 0 | 0 | 1 | 0 | 1 | 1 | 1 | 0 | 1 | 1 |
| 166 | 1 | 1 | 0 | 1 | 0 | 0 | 0 | 0 | 1 | 0 |
| 167 |  |  |  |  |  |  |  |  |  |  |
| 168 | 1 | 1 | 1 | 0 | 0 | 1 | 0 | 1 | 0 | 0 |
| 169 | 1 | 1 | 1 | 0 | 1 | 1 | 1 | 0 | 1 | 0 |
| 170 | 0 | 1 | 0 | 1 | 0 | 1 | 1 | 0 | 0 | 1 |
| 171 | 1 | 1 | 1 | 1 | 0 | 0 | 1 | 1 | 0 | 0 |
| 172 | 1 | 1 | 0 | 1 | 0 | 0 | 0 | 0 | 0 | 0 |
| 173 | 1 | 1 | 1 | 0 | 0 | 1 | 0 | 0 | 0 | 0 |
| 174 | 0 | 1 | 1 | 1 | 0 | 0 | 1 | 0 | 1 | 0 |
| 175 | 0 | 1 | 1 | 1 | 1 | 0 | 1 | 0 | 0 | 0 |
| 176 | 0 | 1 | 1 | 1 | 0 | 1 | 1 | 1 | 1 | 1 |
| 177 | 1 | 0 | 1 | 1 | 0 | 1 | 1 | 0 | 1 | 0 |
| 178 | 1 | 0 | 0 | 0 | 0 | 1 | 1 | 0 | 0 | 0 |
| 179 | 1 | 1 | 1 | 1 | 0 | 0 | 1 | 0 | 1 | 0 |
| 180 | 0 | 0 | 1 | 1 | 0 | 1 | 0 | 1 | 0 | 0 |
| 181 | 1 | 0 | 1 | 1 | 0 | 1 | 1 | 0 | 0 | 0 |
| 182 | 1 | 1 | 1 | 0 | 0 | 1 | 0 | 0 | 0 | 0 |
| 183 | 1 | 1 | 1 | 1 | 0 | 1 | 1 | 1 | 1 | 0 |
| 184 | 1 | 1 | 1 | 0 | 0 | 0 | 1 | 0 | 0 | 0 |
| 185 | 1 | 0 | 1 | 1 | 1 | 1 | 0 | 1 | 1 | 0 |
| 186 | 1 | 0 | 1 | 1 | 0 | 1 | 0 | 1 | 1 | 1 |
| 187 | 0 | 0 | 1 | 1 | 0 | 1 | 0 | 1 | 0 | 0 |
| 188 | 1 | 1 | 1 | 0 | 0 | 0 | 0 | 1 | 0 | 0 |
| 189 | 0 | 1 | 1 | 1 | 0 | 0 | 1 | 0 | 0 | 1 |
| 190 | 1 | 1 | 0 | 0 | 0 | 0 | 1 | 0 | 0 | 0 |
| 192 | 1 | 0 | 0 | 0 | 1 | 0 | 1 | 1 | 1 | 0 |
| 193 | 1 | 0 | 0 | 1 | 0 | 0 | 1 | 1 | 0 | 1 |
| 194 | 0 | 1 | 1 | 0 | 0 | 0 | 1 | 0 | 0 | 1 |
| 195 | 1 | 0 | 1 | 0 | 0 | 0 | 1 | 1 | 1 | 1 |
| 196 | 1 | 0 | 1 | 0 | 0 | 0 | 1 | 0 | 1 | 0 |
| 197 | 0 | 1 | 1 | 1 | 0 | 0 | 1 | 1 | 0 | 0 |
| 198 | 1 | 0 | 0 | 0 | 0 | 1 | 0 | 0 | 0 | 0 |
| 199 | 1 | 1 | 1 | 0 | 0 | 0 | 1 | 1 | 1 | 0 |
| 200 | 0 | 1 | 1 | 1 | 0 | 0 | 0 | 1 | 0 | 0 |
| 201 | 1 | 1 | 1 | 0 | 0 | 0 | 0 | 0 | 0 | 1 |
| 202 | 1 | 1 | 1 | 1 | 0 | 1 | 0 | 1 | 0 | 0 |
| 204 | 1 | 1 | 1 | 1 | 1 | 0 | 0 | 0 | 0 | 1 |
| 205 | 1 | 1 | 0 | 1 | 0 | 0 | 0 | 0 | 0 | 0 |
| 206 | 1 | 0 | 0 | 0 | 0 | 1 | 1 | 1 | 0 | 1 |
| 207 | 1 | 0 | 1 | 0 | 1 | 0 | 1 | 1 | 0 | 0 |
| 208 | 1 | 1 | 1 | 1 | 0 | 1 | 0 | 0 | 0 | 0 |
| 210 | 1 | 0 | 0 | 0 | 0 | 0 | 1 | 0 | 0 | 1 |
| 211 | 1 | 1 | 1 | 0 | 0 | 0 | 0 | 0 | 0 | 1 |
| 212 | 1 | 0 | 0 | 0 | 1 | 0 | 0 | 1 | 1 | 0 |
| 213 | 1 | 1 | 0 | 1 | 0 | 0 | 0 | 1 | 1 | 0 |
| 214 | 0 | 0 | 1 | 1 | 0 | 0 | 0 | 1 | 0 | 0 |
| 216 | 1 | 0 | 0 | 1 | 0 | 1 | 0 | 0 | 0 | 0 |
| 217 | 0 | 0 | 1 | 0 | 1 | 0 | 1 | 1 | 0 | 0 |
| 218 | 0 | 1 | 0 | 0 | 0 | 0 | 0 | 0 | 1 | 0 |
| 219 | 0 | 1 | 1 | 0 | 0 | 0 | 0 | 0 | 0 | 0 |
| 220 | 1 | 1 | 1 | 0 | 0 | 0 | 1 | 0 | 0 | 0 |
| 221 | 0 | 1 | 1 | 0 | 0 | 0 | 0 | 0 | 0 | 0 |
| 222 | 0 | 0 | 0 | 0 | 0 | 1 | 1 | 0 | 0 | 0 |
| 223 | 1 | 1 | 1 | 0 | 0 | 0 | 1 | 1 | 1 | 0 |
| 224 | 1 | 0 | 0 | 1 | 0 | 0 | 1 | 0 | 0 | 0 |
| 225 | 0 | 1 | 1 | 0 | 0 | 0 | 1 | 1 | 0 | 0 |
| 226 | 1 | 1 | 1 | 1 | 1 | 0 | 1 | 0 | 1 | 0 |
| 227 | 1 | 1 | 0 | 1 | 0 | 1 | 0 | 0 | 1 | 1 |
| 228 | 1 | 1 | 0 | 1 | 1 | 1 | 1 | 0 | 0 | 0 |
| 229 | 0 | 1 | 0 | 0 | 1 | 0 | 0 | 0 | 0 | 0 |
| 230 | 0 | 1 | 0 | 0 | 0 | 0 | 0 | 0 | 1 | 1 |
| 231 | 1 | 1 | 0 | 1 | 0 | 0 | 1 | 1 | 0 | 1 |
| 232 | 1 | 1 | 1 | 0 | 0 | 1 | 0 | 1 | 0 | 1 |
| 233 | 0 | 0 | 0 | 1 | 0 | 1 | 0 | 0 | 1 | 0 |
| 234 | 0 | 0 | 0 | 1 | 1 | 1 | 1 | 1 | 1 | 0 |
| 235 | 1 | 1 | 0 | 1 | 0 | 0 | 0 | 0 | 1 | 1 |
| 236 | 0 | 1 | 1 | 0 | 0 | 0 | 1 | 1 | 1 | 0 |
| 237 | 0 | 1 | 0 | 0 | 0 | 0 | 1 | 1 | 0 | 1 |
| 239 | 1 | 1 | 1 | 1 | 0 | 0 | 1 | 1 | 1 | 1 |
| 240 | 1 | 1 | 0 | 1 | 1 | 1 | 1 | 0 | 1 | 0 |
| 241 | 0 | 1 | 0 | 0 | 1 | 1 | 1 | 0 | 0 | 0 |
| 242 | 1 | 0 | 1 | 0 | 0 | 0 | 0 | 1 | 1 | 0 |
| 243 | 0 | 1 | 0 | 1 | 1 | 1 | 1 | 0 | 0 | 0 |
| 244 | 1 | 1 | 1 | 0 | 0 | 1 | 0 | 0 | 0 | 1 |
| 245 | 0 | 0 | 0 | 1 | 0 | 1 | 0 | 0 | 0 | 0 |
| 246 | 1 | 1 | 0 | 1 | 0 | 1 | 1 | 1 | 1 | 0 |
| 247 | 1 | 1 | 1 | 1 | 0 | 1 | 0 | 0 | 0 | 0 |
| 248 | 0 | 0 | 1 | 1 | 0 | 0 | 0 | 0 | 0 | 1 |
| 249 | 1 | 0 | 1 | 0 | 0 | 0 | 0 | 0 | 0 | 0 |
| 250 | 1 | 1 | 0 | 0 | 1 | 1 | 1 | 0 | 0 | 0 |
| 251 | 0 | 0 | 1 | 1 | 0 | 0 | 0 | 1 | 0 | 0 |
| 252 | 0 | 1 | 0 | 0 | 0 | 1 | 1 | 1 | 0 | 0 |
| 254 | 1 | 1 | 1 | 1 | 0 | 1 | 0 | 0 | 0 | 1 |
| 255 | 1 | 1 | 1 | 0 | 0 | 1 | 1 | 0 | 1 | 0 |
| 256 | 0 | 1 | 1 | 0 | 0 | 0 | 0 | 0 | 1 | 0 |
| 257 | 1 | 0 | 1 | 0 | 0 | 1 | 1 | 0 | 1 | 0 |
| 258 | 1 | 1 | 1 | 0 | 0 | 0 | 0 | 0 | 1 | 0 |
| 259 | 1 | 1 | 0 | 0 | 1 | 0 | 1 | 1 | 0 | 0 |
| 260 | 1 | 0 | 0 | 1 | 0 | 1 | 0 | 0 | 0 | 0 |
| 262 | 0 | 1 | 1 | 0 | 1 | 0 | 1 | 1 | 1 | 1 |
| 263 | 1 | 0 | 0 | 1 | 0 | 0 | 1 | 0 | 0 | 0 |
| 264 | 1 | 1 | 0 | 1 | 0 | 0 | 1 | 0 | 0 | 1 |
| 265 | 0 | 1 | 0 | 0 | 0 | 1 | 0 | 0 | 1 | 0 |
| 266 | 1 | 0 | 1 | 0 | 1 | 0 | 0 | 0 | 1 | 0 |
| 267 | 1 | 1 | 1 | 0 | 0 | 0 | 1 | 0 | 1 | 1 |
| 268 | 1 | 1 | 1 | 1 | 0 | 0 | 1 | 0 | 1 | 0 |
| 269 | 1 | 0 | 0 | 1 | 0 | 0 | 1 | 1 | 1 | 0 |
| 270 | 1 | 1 | 0 | 1 | 0 | 0 | 0 | 1 | 1 | 0 |
| 272 | 1 | 0 | 0 | 1 | 0 | 0 | 0 | 0 | 0 | 0 |
| 273 | 0 | 1 | 1 | 1 | 0 | 0 | 0 | 0 | 1 | 0 |
| 274 | 1 | 1 | 0 | 0 | 0 | 1 | 0 | 1 | 1 | 1 |
| 275 | 1 | 1 | 1 | 1 | 1 | 0 | 1 | 0 | 0 | 0 |
| 276 | 1 | 1 | 1 | 1 | 0 | 0 | 1 | 1 | 0 | 0 |
| 277 | 1 | 0 | 1 | 0 | 0 | 0 | 1 | 0 | 1 | 0 |
| 278 | 1 | 1 | 1 | 0 | 0 | 1 | 1 | 0 | 0 | 0 |
| 279 | 0 | 0 | 1 | 0 | 0 | 0 | 1 | 0 | 0 | 0 |
| 280 | 1 | 0 | 1 | 1 | 0 | 1 | 1 | 0 | 0 | 0 |
| 281 | 1 | 1 | 1 | 0 | 0 | 0 | 0 | 0 | 1 | 0 |
| 283 | 1 | 1 | 0 | 1 | 0 | 0 | 0 | 1 | 0 | 1 |
| 284 | 0 | 1 | 0 | 0 | 0 | 1 | 0 | 0 | 0 | 0 |
| 286 | 1 | 0 | 0 | 0 | 1 | 1 | 1 | 0 | 1 | 0 |
| 287 | 1 | 1 | 1 | 1 | 1 | 0 | 0 | 0 | 1 | 1 |
| 288 | 0 | 0 | 0 | 1 | 0 | 0 | 1 | 0 | 0 | 1 |
| 289 | 1 | 0 | 1 | 0 | 0 | 0 | 1 | 0 | 0 | 1 |
| 290 | 1 | 1 | 1 | 0 | 0 | 0 | 0 | 0 | 0 | 1 |
| 291 | 0 | 0 | 1 | 0 | 0 | 0 | 0 | 0 | 0 | 0 |
| 292 | 1 | 1 | 1 | 1 | 0 | 0 | 1 | 0 | 0 | 1 |
| 293 | 1 | 1 | 0 | 0 | 1 | 1 | 1 | 1 | 0 | 1 |
| 294 | 0 | 0 | 1 | 0 | 0 | 0 | 0 | 1 | 1 | 0 |
| 295 | 0 | 1 | 0 | 0 | 0 | 0 | 0 | 1 | 0 | 0 |
| 296 | 1 | 1 | 0 | 0 | 0 | 1 | 1 | 1 | 1 | 0 |
| 297 | 0 | 1 | 1 | 1 | 1 | 0 | 0 | 0 | 1 | 1 |
| 298 | 1 | 1 | 1 | 1 | 0 | 0 | 0 | 1 | 0 | 0 |
| 299 | 1 | 0 | 0 | 1 | 0 | 1 | 0 | 0 | 1 | 0 |
| 300 | 0 | 0 | 0 | 1 | 0 | 1 | 0 | 0 | 0 | 0 |
| 301 | 0 | 1 | 1 | 0 | 0 | 0 | 0 | 1 | 0 | 0 |
| 302 | 1 | 1 | 0 | 1 | 1 | 0 | 0 | 0 | 1 | 0 |
| 303 | 1 | 0 | 1 | 0 | 0 | 1 | 1 | 0 | 0 | 0 |
| 304 | 1 | 1 | 1 | 1 | 0 | 0 | 1 | 0 | 0 | 1 |
| 305 | 0 | 1 | 1 | 1 | 0 | 0 | 0 | 0 | 0 | 0 |
| 306 | 0 | 0 | 1 | 1 | 0 | 1 | 1 | 1 | 0 | 0 |
| 307 | 0 | 1 | 0 | 1 | 0 | 0 | 0 | 1 | 0 | 0 |
| 308 | 1 | 0 | 1 | 1 | 0 | 1 | 1 | 1 | 1 | 0 |
| 309 | 0 | 1 | 0 | 1 | 0 | 1 | 1 | 1 | 1 | 1 |
| 310 | 0 | 0 | 0 | 1 | 0 | 0 | 1 | 1 | 1 | 0 |
| 311 | 0 | 1 | 1 | 1 | 0 | 0 | 0 | 0 | 1 | 1 |
| 312 | 0 | 0 | 1 | 0 | 0 | 0 | 0 | 0 | 0 | 0 |
| 313 | 1 | 0 | 1 | 0 | 1 | 0 | 0 | 1 | 1 | 0 |
| 314 | 0 | 1 | 1 | 0 | 0 | 0 | 0 | 0 | 1 | 1 |
| 315 | 0 | 1 | 1 | 1 | 0 | 1 | 0 | 0 | 0 | 1 |
| 316 | 1 | 1 | 1 | 0 | 0 | 1 | 1 | 0 | 0 | 0 |
| 317 | 1 | 1 | 0 | 0 | 0 | 1 | 1 | 0 | 0 | 0 |
| 318 | 1 | 1 | 1 | 0 | 0 | 0 | 1 | 0 | 0 | 1 |
| 319 | 1 | 0 | 1 | 0 | 0 | 0 | 1 | 0 | 1 | 0 |
| 320 | 1 | 1 | 1 | 0 | 0 | 0 | 0 | 1 | 1 | 1 |
| 321 | 1 | 1 | 1 | 1 | 0 | 0 | 1 | 0 | 0 | 0 |
| 322 | 0 | 0 | 1 | 1 | 0 | 0 | 1 | 0 | 1 | 0 |
| 323 | 0 | 1 | 1 | 1 | 0 | 1 | 0 | 0 | 0 | 0 |
| 324 | 1 | 1 | 0 | 1 | 0 | 1 | 0 | 0 | 0 | 0 |
| 325 | 1 | 1 | 1 | 1 | 0 | 1 | 1 | 1 | 1 | 0 |
| 326 | 1 | 0 | 0 | 0 | 0 | 0 | 0 | 1 | 1 | 0 |
| 327 | 0 | 1 | 1 | 0 | 0 | 1 | 1 | 0 | 0 | 1 |
| 328 | 1 | 1 | 1 | 0 | 0 | 1 | 0 | 1 | 1 | 1 |
| 329 | 1 | 1 | 0 | 1 | 1 | 1 | 0 | 0 | 1 | 0 |
| 330 | 1 | 1 | 1 | 1 | 0 | 0 | 1 | 0 | 1 | 0 |
| 331 | 1 | 1 | 1 | 0 | 1 | 0 | 0 | 0 | 0 | 0 |
| 332 | 1 | 1 | 0 | 1 | 0 | 0 | 1 | 1 | 0 | 0 |
| 333 | 0 | 1 | 1 | 0 | 0 | 0 | 0 | 0 | 0 | 1 |
| 334 | 1 | 1 | 0 | 1 | 1 | 0 | 1 | 1 | 0 | 0 |
| 335 | 0 | 1 | 0 | 0 | 1 | 1 | 1 | 1 | 0 | 0 |
| 336 | 1 | 0 | 1 | 0 | 1 | 0 | 0 | 0 | 0 | 0 |
| 337 | 1 | 1 | 1 | 0 | 0 | 1 | 0 | 0 | 0 | 0 |
| 339 | 0 | 1 | 1 | 0 | 0 | 0 | 1 | 0 | 1 | 0 |
| 340 | 0 | 0 | 1 | 0 | 0 | 0 | 0 | 0 | 0 | 0 |
| 341 | 1 | 1 | 0 | 0 | 0 | 0 | 0 | 0 | 1 | 0 |
| 342 | 1 | 1 | 0 | 0 | 0 | 0 | 1 | 0 | 1 | 1 |
| 343 | 0 | 1 | 1 | 1 | 0 | 1 | 1 | 0 | 1 | 0 |
| 344 | 1 | 0 | 0 | 0 | 0 | 0 | 1 | 0 | 1 | 0 |
| 345 | 1 | 1 | 1 | 0 | 0 | 0 | 0 | 1 | 0 | 0 |
| 346 | 1 | 1 | 0 | 1 | 1 | 0 | 1 | 0 | 0 | 0 |
| 347 | 1 | 0 | 0 | 0 | 0 | 0 | 1 | 0 | 1 | 0 |
| 348 | 1 | 1 | 0 | 0 | 0 | 0 | 1 | 1 | 0 | 0 |
| 349 | 0 | 1 | 1 | 0 | 1 | 0 | 1 | 1 | 0 | 0 |
| 350 | 1 | 0 | 0 | 1 | 0 | 1 | 1 | 0 | 1 | 0 |
| 351 | 1 | 1 | 1 | 0 | 0 | 0 | 0 | 0 | 0 | 1 |
| 352 | 1 | 1 | 1 | 0 | 1 | 1 | 1 | 0 | 0 | 0 |
| 353 | 0 | 1 | 1 | 1 | 0 | 0 | 1 | 0 | 0 | 1 |
| 354 | 1 | 0 | 1 | 0 | 0 | 0 | 1 | 1 | 1 | 0 |
| 355 | 0 | 1 | 1 | 0 | 0 | 0 | 1 | 0 | 0 | 0 |
| 356 | 0 | 1 | 1 | 1 | 0 | 0 | 0 | 0 | 1 | 0 |
| 357 | 0 | 0 | 0 | 1 | 0 | 1 | 1 | 0 | 1 | 0 |
| 358 | 0 | 1 | 0 | 1 | 0 | 0 | 0 | 1 | 0 | 0 |
| 359 | 0 | 1 | 1 | 1 | 1 | 1 | 0 | 0 | 1 | 0 |
| 360 | 0 | 1 | 1 | 1 | 0 | 1 | 0 | 1 | 0 | 1 |
| 361 | 0 | 1 | 1 | 0 | 1 | 0 | 0 | 0 | 0 | 1 |
| 362 | 0 | 0 | 1 | 0 | 0 | 0 | 0 | 0 | 1 | 1 |
| 363 | 0 | 0 | 0 | 1 | 0 | 0 | 0 | 0 | 0 | 1 |
| 364 | 1 | 0 | 1 | 0 | 1 | 0 | 1 | 0 | 1 | 0 |
| 365 | 1 | 1 | 0 | 1 | 0 | 0 | 1 | 0 | 1 | 0 |
| 366 |  |  | 0 | 0 | 0 | 0 | 0 | 0 | 1 | 1 |
| 367 | 1 | 1 | 1 | 1 | 0 | 0 | 1 | 1 | 0 | 1 |
| 368 | 1 | 1 | 1 | 1 | 0 | 1 | 0 | 0 | 1 | 1 |
| 369 | 0 | 0 | 0 | 0 | 0 | 0 | 0 | 0 | 0 | 1 |
| 370 | 1 | 0 | 1 | 0 | 0 | 0 | 0 | 1 | 1 | 0 |
| 371 | 0 | 0 | 1 | 1 | 0 | 0 | 1 | 0 | 1 | 0 |
| 372 | 0 | 0 | 0 | 1 | 0 | 0 | 1 | 0 | 1 | 0 |
| 373 | 1 | 1 | 1 | 0 | 0 | 0 | 0 | 0 | 0 | 0 |
| 374 | 1 | 1 | 1 | 0 | 0 | 1 | 1 | 1 | 0 | 1 |
| 375 | 0 | 1 | 1 | 1 | 0 | 0 | 1 | 0 | 0 | 1 |
| 376 | 0 | 1 | 1 | 1 | 1 | 0 | 1 | 0 | 0 | 0 |
| 377 | 0 | 1 | 1 | 1 | 0 | 0 | 0 | 1 | 1 | 0 |
| 378 | 1 | 1 | 1 | 0 | 0 | 0 | 0 | 1 | 0 | 1 |
| 379 | 0 | 0 | 1 | 0 | 0 | 1 | 1 | 0 | 1 | 0 |
| 380 | 1 | 0 | 1 | 0 | 1 | 0 | 0 | 0 | 1 | 0 |
| 381 | 1 | 0 | 1 | 1 | 1 | 0 | 1 | 0 | 1 | 0 |
| 382 | 1 | 0 | 1 | 0 | 0 | 0 | 1 | 0 | 0 | 0 |
| 383 | 1 | 0 | 1 | 1 | 1 | 0 | 1 | 1 | 0 | 0 |
| 384 | 1 | 0 | 1 | 1 | 0 | 0 | 1 | 0 | 1 | 0 |
| 385 | 0 | 0 | 1 | 1 | 0 | 0 | 0 | 0 | 1 | 0 |
| 386 | 0 | 1 | 0 | 1 | 0 | 0 | 0 | 1 | 1 | 0 |
| 387 | 0 | 1 | 1 | 1 | 0 | 1 | 1 | 0 | 0 | 1 |
| 388 | 0 | 1 | 1 | 1 | 0 | 0 | 1 | 0 | 1 | 0 |
| 389 | 1 | 1 | 1 | 0 | 0 | 1 | 0 | 0 | 0 | 0 |
| 390 | 0 | 0 | 0 | 1 | 0 | 0 | 1 | 0 | 1 | 1 |
| 391 | 1 | 1 | 1 | 1 | 0 | 0 | 1 | 0 | 0 | 1 |
| 392 | 0 | 1 | 1 | 0 | 0 | 1 | 1 | 0 | 1 | 0 |
| 393 | 1 | 1 | 1 | 0 | 0 | 0 | 0 | 0 | 1 | 0 |
| 394 | 0 | 0 | 1 | 0 | 0 |  | 1 | 1 | 0 | 0 |
| 395 | 0 | 1 | 0 | 0 | 0 | 0 | 0 | 1 | 1 | 0 |
| 396 | 1 | 0 | 0 | 1 | 0 | 0 | 0 | 1 | 1 | 0 |
| 397 | 0 | 0 | 0 | 1 | 0 | 0 | 1 | 0 | 0 | 0 |
| 398 | 1 | 1 | 0 | 0 | 0 | 1 | 1 | 1 | 0 | 0 |
| 399 | 1 | 1 | 0 | 1 | 1 | 1 | 0 | 1 | 0 | 0 |
| 400 | 1 | 1 | 1 | 0 | 0 | 0 | 1 | 1 | 1 | 0 |
| 402 | 0 | 0 | 1 | 1 | 0 | 1 | 1 | 0 | 0 | 0 |
| 403 | 0 | 0 | 0 | 1 | 1 | 0 | 1 | 1 | 0 | 1 |
| 404 | 1 | 1 | 1 | 0 | 0 | 1 | 1 | 0 | 0 | 0 |
| 405 | 0 | 0 | 1 | 0 | 1 | 0 | 1 | 0 | 1 | 0 |
| 406 | 1 | 1 | 1 | 1 | 1 | 1 | 1 | 0 | 1 | 0 |
| 407 | 1 | 0 | 0 | 1 | 0 | 1 | 0 | 0 | 0 | 0 |
| 408 | 0 | 1 | 0 | 1 | 0 | 0 | 1 | 0 | 0 | 0 |
| 409 | 1 | 1 | 0 | 1 | 0 | 0 | 0 | 0 | 0 | 0 |
| 410 | 0 | 1 | 1 | 1 | 0 | 0 | 0 | 1 | 0 | 0 |
| 411 | 0 | 1 | 1 | 1 | 0 | 1 | 0 | 1 | 1 | 1 |
| 412 | 1 | 0 | 1 | 1 | 0 | 1 | 1 | 0 | 0 | 0 |
| 413 | 0 | 1 | 1 | 1 | 0 | 0 | 0 | 0 | 1 | 0 |
| 414 | 0 | 1 | 0 | 0 | 0 | 0 | 0 | 0 | 1 | 0 |
| 415 | 0 | 1 | 1 | 0 | 0 | 0 | 1 | 0 | 1 | 1 |
| 416 | 0 | 1 | 1 | 0 | 0 | 0 | 1 | 1 | 1 | 0 |
| 417 | 1 | 0 | 1 | 1 | 0 | 1 | 0 | 0 | 1 | 0 |
| 418 | 1 | 1 | 1 | 1 | 1 | 0 | 1 | 0 | 0 | 0 |
| 419 | 1 | 1 | 1 | 0 | 0 | 0 | 1 | 0 | 1 | 1 |
| 420 | 0 | 1 | 1 | 1 | 0 | 1 | 1 | 1 | 0 | 1 |
| 421 | 1 | 1 | 1 | 0 | 0 | 0 | 0 | 0 | 0 | 1 |
| 422 | 1 | 0 | 1 | 1 | 0 | 0 | 1 | 1 | 1 | 1 |
| 423 | 0 | 0 | 1 | 0 | 1 | 1 | 0 | 0 | 0 | 0 |
| 424 | 1 | 0 | 1 | 1 | 0 | 0 | 0 | 0 | 0 | 1 |
| 425 | 1 | 0 | 0 | 1 | 0 | 0 | 1 | 0 | 0 | 1 |
| 426 | 1 | 1 | 1 | 1 | 0 | 1 | 1 | 1 | 0 | 0 |
| 427 | 0 | 1 | 1 | 0 | 0 | 1 | 1 | 0 | 1 | 0 |
| 428 | 1 | 0 | 1 | 0 | 0 | 0 | 0 | 0 | 0 | 1 |
| 429 | 1 | 0 | 1 | 1 | 0 | 1 | 1 | 0 | 1 |  |
| 430 | 1 | 1 | 0 | 0 | 0 | 0 | 0 | 1 | 0 | 0 |
| 431 | 0 | 0 | 1 | 0 | 1 | 1 | 1 | 0 | 1 | 0 |
| 432 | 1 | 1 | 1 | 0 |  | 0 | 0 | 0 | 1 | 0 |
| 433 | 1 | 0 | 1 | 0 | 0 | 0 | 0 | 0 | 1 | 0 |
| 434 | 0 | 1 | 1 | 0 | 0 | 0 | 0 | 1 | 1 | 0 |
| 435 | 1 | 0 | 1 | 1 | 0 | 0 | 0 | 1 | 0 | 0 |
| 436 | 1 | 1 | 0 | 1 | 0 | 0 | 0 | 1 | 0 | 1 |
| 437 | 1 | 0 | 0 | 0 | 0 | 0 | 1 | 1 | 1 |  |
| 439 | 1 | 0 | 0 | 1 | 0 | 1 | 0 | 1 | 1 |  |
| 440 | 1 | 1 | 1 | 1 | 0 | 0 | 0 | 0 | 0 |  |
| 442 | 1 | 1 | 1 | 0 | 0 | 0 | 0 | 1 | 0 | 0 |
| 443 | 1 | 1 | 0 | 0 | 1 | 1 | 1 | 1 | 1 | 0 |
| 444 | 0 | 0 | 1 | 1 | 1 | 0 | 0 | 0 | 1 | 0 |
| 445 | 1 | 1 | 1 | 1 | 0 | 0 | 1 | 0 | 0 | 0 |
| 446 | 1 | 0 | 1 | 1 | 1 | 0 | 1 | 1 | 1 | 0 |
| 447 | 1 | 1 | 1 | 1 | 0 | 0 | 1 | 0 | 1 | 0 |
| 448 | 1 | 0 | 1 | 0 | 0 | 0 | 1 | 0 | 0 | 1 |
| 449 | 0 | 0 | 0 | 0 | 0 | 0 | 0 | 1 | 0 | 1 |
| 450 | 1 | 0 | 0 | 1 | 0 | 0 | 1 | 0 | 1 | 0 |
| 451 | 1 | 0 | 0 | 1 | 0 | 0 | 1 | 1 | 1 | 0 |
| 452 | 1 | 1 | 0 | 0 | 0 | 0 | 0 | 0 | 0 | 0 |
| 454 | 0 | 1 | 0 | 0 | 0 | 0 | 1 | 0 | 0 | 0 |
| 455 | 0 | 0 | 0 | 0 | 0 | 0 | 0 | 0 | 0 | 0 |
| 456 | 1 | 1 | 0 | 1 | 0 | 0 | 1 | 1 | 0 | 1 |
| 457 | 0 | 1 | 1 | 0 | 0 | 0 | 0 | 0 | 1 | 0 |
| 458 | 0 | 0 | 1 | 0 | 0 | 0 | 0 | 0 | 1 | 0 |
| 459 | 1 | 0 | 1 | 1 | 0 | 0 | 1 | 1 | 0 | 0 |
| 460 | 1 | 0 | 1 | 0 | 0 | 1 | 0 | 0 | 1 | 0 |
| 461 | 1 | 1 | 0 | 0 | 0 | 0 | 1 | 0 | 1 | 0 |
| 462 | 1 | 1 | 0 | 0 | 0 | 1 | 0 | 1 | 0 | 0 |
| 464 | 1 | 0 | 1 | 1 | 0 | 0 | 1 | 0 | 0 | 0 |
| 465 | 0 | 1 | 1 | 0 | 0 | 1 | 1 | 0 | 0 | 1 |
| 466 | 0 | 0 | 0 | 1 | 0 | 1 | 0 | 1 | 0 | 1 |
| 468 | 1 | 1 | 1 | 0 | 0 | 1 | 1 | 0 | 1 | 0 |
| 469 | 1 | 1 | 0 | 0 | 0 | 0 | 1 | 0 | 0 | 0 |
| 470 | 1 | 1 | 1 | 0 | 0 | 0 | 0 | 1 | 0 | 0 |
| 471 | 1 | 1 | 1 | 0 | 0 | 0 | 0 | 0 | 0 | 0 |
| 472 | 1 | 0 | 1 | 1 | 0 | 0 | 0 | 0 | 0 | 1 |
| 473 | 1 | 1 | 1 | 0 | 0 | 0 | 0 | 0 | 0 | 0 |
| 474 | 0 | 1 | 1 | 0 | 0 | 0 | 1 | 0 | 1 | 0 |
| 475 | 1 | 1 | 0 | 1 | 1 | 0 | 1 | 1 | 0 | 0 |
| 476 |  |  |  |  |  |  |  |  |  |  |
| 477 |  |  |  |  |  |  |  |  |  |  |
| 478 |  |  |  |  |  |  |  |  |  |  |
| 479 |  |  |  |  |  |  |  |  |  |  |
